# Histone H3 Lysine 27 demethylases Jmjd3 and Utx are required for T-cell differentiation

**DOI:** 10.1038/ncomms9152

**Published:** 2015-09-02

**Authors:** Sugata Manna, Jong Kyong Kim, Catherine Baugé, Margaret Cam, Yongmei Zhao, Jyoti Shetty, Melanie S. Vacchio, Ehydel Castro, Bao Tran, Lino Tessarollo, Rémy Bosselut

**Affiliations:** 1 Laboratory of Immune Cell Biology, Center for Cancer Research, National Cancer Institute, National Institutes of Health, Bethesda, Maryland 20892, USA; 2 Collaborative Bioinformatics Resource, Center for Cancer Research, National Cancer Institute, National Institutes of Health, Bethesda, Maryland 20892, USA; 3 Leidos Biomedical Research, Frederick National Laboratory for Cancer Research, Frederick, Maryland 21702, USA; 4 Mouse Cancer Genetics Program, Center for Cancer Research, National Cancer Institute, National Institutes of Health, Frederick, Maryland 21702, USA

## Abstract

Although histone H3 lysine 27 trimethylation (H3K27Me3) is associated with gene silencing, whether H3K27Me3 demethylation affects transcription and cell differentiation *in vivo* has remained elusive. To investigate this, we conditionally inactivated the two H3K27Me3 demethylases, Jmjd3 and Utx, in non-dividing intrathymic CD4^+^ T-cell precursors. Here we show that both enzymes redundantly promote H3K27Me3 removal at, and expression of, a specific subset of genes involved in terminal thymocyte differentiation, especially *S1pr1*, encoding a sphingosine-phosphate receptor required for thymocyte egress. Thymocyte expression of S1pr1 was not rescued in Jmjd3- and Utx-deficient male mice, which carry the catalytically inactive Utx homolog Uty, supporting the conclusion that it requires H3K27Me3 demethylase activity. These findings demonstrate that Jmjd3 and Utx are required for T-cell development, and point to a requirement for their H3K27Me3 demethylase activity in cell differentiation.

## Abstract (web summary)

Histone post-translational modifications such as the trimethylation of histone H3 at lysine 27 (H3K27Me3), affect DNA accessibility and transcription. Here, the authors show that Jmjd3 and Utx, two H3K27Me3 demethylases, are important for regulating the expression of genes involved in terminal thymocyte differentiation.

## Graphical abstract

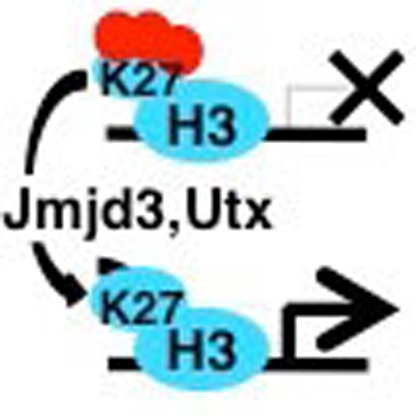

Lineage differentiation during metazoan development is mediated by cell-specific transcriptional programs directing gene expression. The control of such transcriptional ‘circuits' involves transcription factors that directly or indirectly bind specific DNA sequences, and locus-specific modifications of DNA or histones. Although much progress has been made in identifying transcription factors driving cell differentiation, including during T-cell development in the thymus^1^,^2^, the role of DNA or histone modifiers is less well understood.

Much attention has focused on histone post-translational modifications, including methylation, acetylation and ubiquitination^3^,^4^, which affect DNA accessibility and recruitment of specific transcriptional activators or repressors. The trimethylation of histone H3 lysine 27 (H3K27Me3) is of specific interest because it promotes lineage-specific gene silencing in embryonic stem cells and is therefore thought to be important to maintain multipotency^5^,^6^,^7^,^8^. H3 K27 is methylated by Ezh2, a methyl transferase part of Polycomb-complex 2 (PRC2), or the related molecule Ezh1. H3K27Me3 accumulation at promoter sites promotes silencing at least in part through recruitment of Polycomb-complex 1 (PRC1) components^5^,^6^,^7^,^8^.

Even though gene-targeted clearance of H3K27Me3 is thought to be essential for the emergence of lineage-specific gene expression during development, the mechanisms governing the removal of the mark are not well understood. The discovery of H3K27 demethylases Jmjd3 and Utx has suggested that active demethylation contributes to the depletion of H3K27Me3 and to initiation of lineage-specific gene expression^9^,^10^,^11^,^12^,^13^. However, whereas both molecules are necessary during mouse embryonic or postnatal development^14^,^15^,^16^,^17^,^18^,^19^, it has remained unclear whether such biological functions involve their demethylase activity. Indeed, a Y-chromosome-encoded homolog of Utx, Uty, which lacks demethylase activity, rescues Utx functions in Utx-deficient male embryonic stem cells and embryos^14^,^16^,^17^,^18^, in which its expression largely mirrors that of Utx^16^,^20^. Similarly, a Utx mutant engineered to lack demethylase activity rescues the development of female Utx-deficient embryos^17^,^18^. Strikingly, Uty rescues the development of embryos lacking both Utx and Jmjd3 (ref. 21). Thus, the contribution of Jmjd3 and Utx demethylase activity to their biological functions has remained elusive.

We thought that assessing the role of these molecules in T-cell development would help address this critical question. In developing thymocytes and in effector T-cells, gene-specific levels of H3K27 methylation are developmentally controlled and removal of the tri-methyl mark is associated with induction of gene expression, in keeping with the idea that the mark promotes gene repression^22^,^23^,^24^,^25^. In the thymus, H3K27Me3 developmental removal has been observed at the promoter of the gene (*Zbtb7b*) encoding the CD4^+^-lineage-specific transcription factor Thpok^26^, a molecule required for the development of CD4^+^ T-cells from CD4^+^CD8^+^ (‘double positive', DP) thymic precursors^27^. Furthermore, the differentiation of DP thymocytes into CD4^+^-lineage cells is not associated with cell division, suggesting that an active H3K27Me3 removal mechanism is required to clear the mark; in contrast, rapidly proliferating cells can conceivably ‘dilute' the mark during cell division.

By examining the role of Jmjd3 and Utx in non-dividing DP and CD4^+^ and CD8^+^ ‘single-positive' thymocytes, the present study shows that these enzymes are redundantly required for H3K27Me3 homeostasis and gene expression. They are needed for the removal of H3K27Me3 at, and expression of, multiple promoters characteristic of mature thymocytes and T-cells, in particular *S1pr1*, a gene needed for thymocyte egress. As a result, Jmjd3 and Utx are required for the generation of a normal T-cell repertoire. Importantly, *S1pr1* expression was not rescued by Uty in male cells lacking both Jmjd3 and Utx, supporting the idea that it requires Jmjd3- or Utx-catalyzed H3K27Me3 demethylation at its promoter. These findings demonstrate that Jmjd3 and Utx are required for T-cell differentiation.

## Results

### Jmjd3 and Utx are important for T-cell development

To evaluate the role of Utx and Jmjd3 in T-cell development, we inactivated the genes encoding these enzymes (*Kdm6a* and *Kdm6b*, respectively called *Utx* and *Jmjd3* hereafter). Because germline disruption of either gene causes embryonic or neonatal death^14^,^16^,^17^,^18^,^28^, we used *Cd4*-Cre to target DP thymocytes for deletion of *Jmjd3*^f^ and *Utx*^f^ conditional alleles (Supplementary Fig. 1a,b), in which LoxP sequences flank exons encoding the catalytic JmjC domain^29^. We verified deletion of floxed sequences in thymocytes from *Jmjd3*^f/f^ and *Utx*^f/f^ *Cd4*-Cre mice (called *Jmjd3*- and *Utx*-deficient or -KO hereafter) by genomic DNA PCR, and loss of protein expression by immunoblotting (Supplementary Fig. 2a–c).

**Figure 1:**
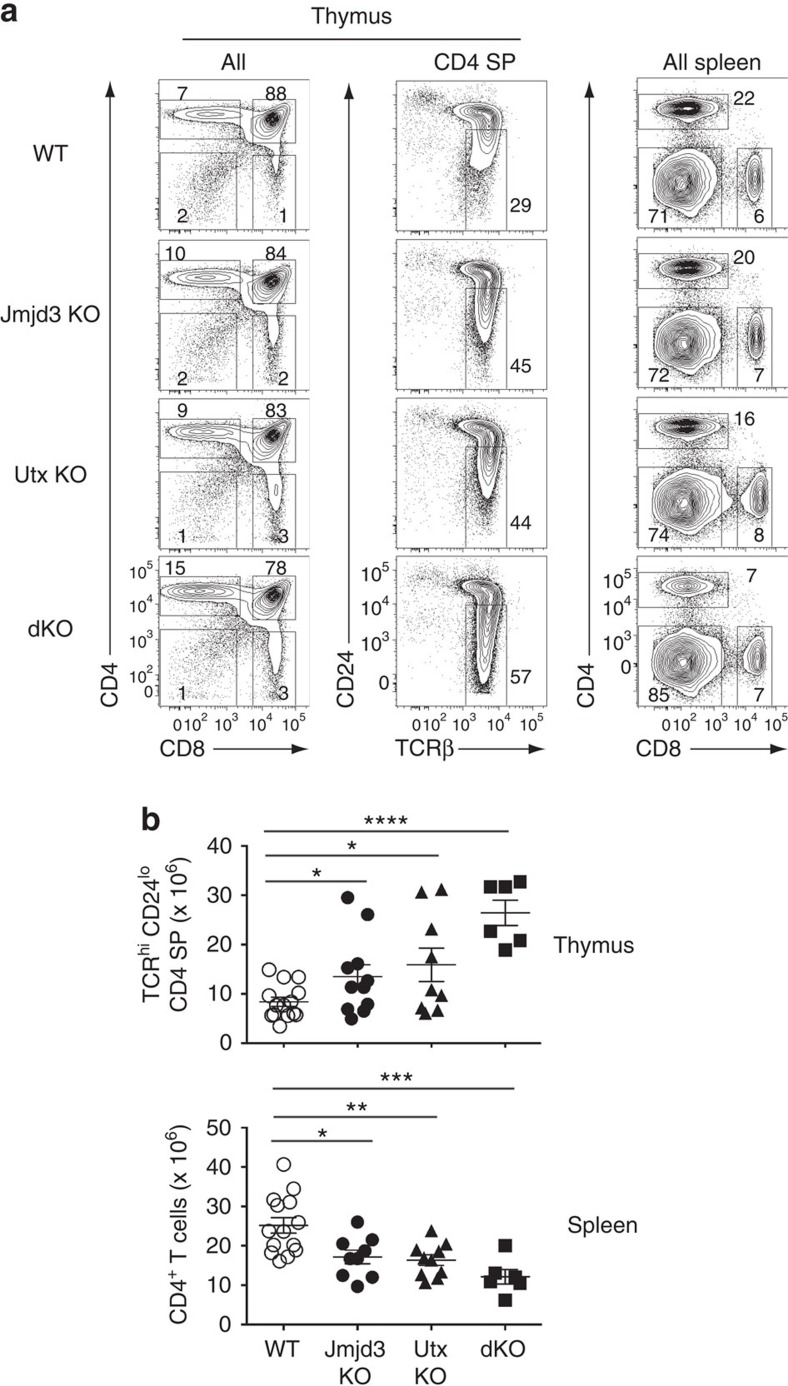
T-cell development in the absence of H3K27 demethylases. (**a**) Contour plots of CD4 and CD8 expression in thymocytes (left) or splenocytes (right), and expression of TCRβ and CD24 on CD4 SP thymocytes (middle) from wild-type (WT), *Jmjd3*^*f/f*^*Cd4-Cre* (Jmjd3 KO), *Utx*^*f/f*^*Cd4-Cre* (Utx KO) or *Jmjd3*^*f/f*^ *Utx*^*f/f*^*Cd4-Cre* (dKO) mice. Numbers adjacent to outlined areas indicate percentage T-cells. Numbers of mice for each genotype indicated in (**b**). The gate (middle column) defines mature (TCR^hi^ CD24^lo^) CD4 SP thymocytes. Data is representative of 10 (Jmjd3 KO), 9 (Utx KO) and 5 (dKO) separate experiments. (**b**) Dot plots show absolute cell numbers of mature (TCR^hi^ CD24^lo^) CD4 SP thymocytes or spleen CD4^+^ T-cells from mice in (**a**). The statistical significance was determined by unpaired *t*-test (two-tailed). *: *P*<0.05, **: *P*<0.01, ***: *P*<0.001, ****: *P*<0.0001. Each symbol represents one individual mouse. Error bars indicate s.d.

CD4 and CD8 SP thymocytes developed in mice with *Cd4*-Cre-mediated deletion of *Jmjd3*, *Utx* or both (dKO thereafter) (Fig. 1a). However, we noted increased numbers of CD4 SP thymocytes; most of them were mature post-selection cells, characterized by high-level T-cell antigen receptor (TCR) and low-level CD24 expression (TCR^hi^ CD24^lo^, Fig. 1a,b). Increased thymocyte numbers contrasted with reduced numbers of total and naive CD4^+^ T-cells in the spleen (Fig. 1a,b and Supplementary Fig. 3a). Because the developmental block was incomplete even in double mutant mice, and because effects on CD8^+^ T-cell differentiation were not as pronounced (Supplementary Fig. 3b,c), we considered that earlier disruption of *Jmjd3* and *Utx* might have a greater impact on thymocyte development. Consequently, we inactivated *Jmjd3* and *Utx* using *Lck*-Cre, which becomes active in earlier T-cell progenitors than *Cd4*-Cre. *Lck*-Cre disruption of both genes resulted in an accumulation of mature (TCR^hi^ CD24^lo^) CD4 SP thymocytes and a CD4^+^ lymphopenia similar to those seen with *Cd4*-Cre disruption (Supplementary Fig. 3d,e). Of note, the total number of thymocytes was not reduced by *Lck*-Cre-mediated deletion of *Jmjd3* and *Utx* (Supplementary Fig. 3e, top graph), indicating that neither enzyme was required for cell proliferation during early T-cell development. These experiments suggested that Jmjd3 and Utx are specifically important for thymocyte differentiation beyond the DP stage, and we further explored their function using *Cd4*-Cre-mediated disruption.

**Figure 2:**
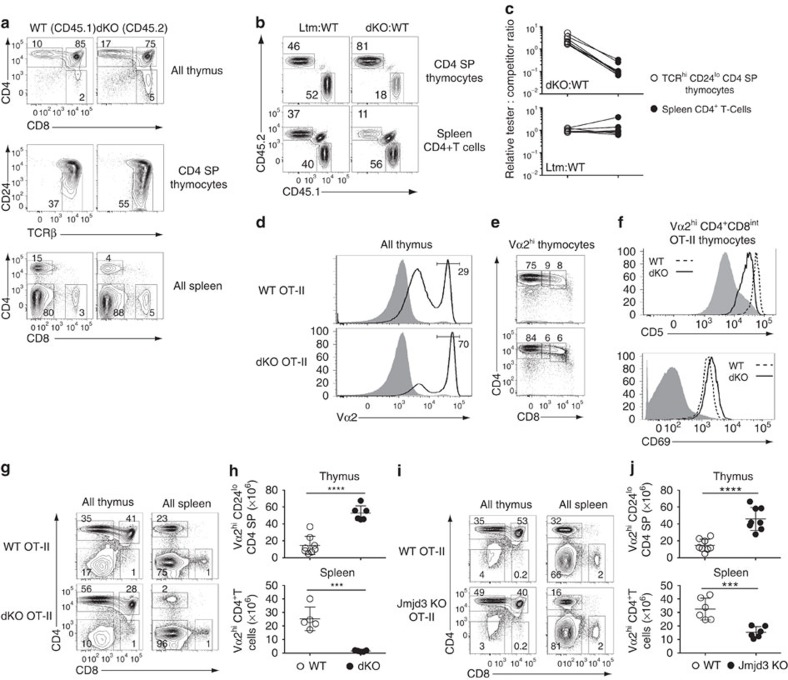
Jmjd3 and Utx are required for thymocyte maturation. (**a**) Expression of CD4 and CD8 on thymocytes (top) or splenocytes (bottom), and expression of TCRβ and CD24 on CD4 SP thymocytes (middle) from dKO (CD45.2) and wild-type competitor (CD45.1) derived cells in mixed bone marrow chimeras. (**b**) Contour plots of CD45.1 and CD45.2 expression on CD4 SP thymocytes (top) or spleen CD4^+^T-cells (bottom) from chimeras made from CD45.1 wild-type and CD45.2 dKO (right) or littermate controls (left) donors. Note the remaining cells of host origin (CD45.1^+^CD45.2^+^) in the spleen. (**c**) Line graphs indicate relative chimerism (defined as the ratio of CD45.2 ‘tester' to CD45.1 competitor cells, expressed relative to that same ratio in DP thymocytes) in mature (TCR^hi^CD24^lo^) CD4 SP thymocytes (left) and spleen CD4^+^T-cells (right). Data are from eight chimeric mice for each donor cell mix, generated in three distinct transplantations and analysed in eight separate experiments (**a**–**c**). (**d**) Overlaid histograms show Vα2 expression in wild-type (top) and dKO (bottom) thymocytes carrying the OT-II TCR transgene (plain line) or in DP thymocytes from non-transgenic wild-type mice (grey shaded). (**e**) CD4 and CD8 expression on Vα2^hi^ thymocytes (as gated in (**d**)) from OT-II transgenic wild-type and dKO mice. (**f**) Overlaid histograms show expression of CD5 (top) and CD69 (bottom) on wild type (dotted line) and dKO (plain line) OT-II transgenic Vα2^hi^CD4^+^CD8^int^ thymocytes (middle gate in (**e**)). Grey shaded histograms indicate expression of each molecule in DP thymocytes from non-transgenic wild-type mice. Data are representative of six (**d**,**e**), or three (**f**) distinct experiments. (**g**–**j**) Contour plots of CD4 and CD8 expression on total thymocytes or splenocytes (**g**,**i**) and absolute numbers of mature thymocytes or spleen Vα2^hi^ CD4^+^ T-cells (**h**,**j**) from wild-type (WT) and dKO (**g**,**h**) or Jmjd3 KO (**i**,**j**) OT-II transgenic mice. Data is from six (**g**,**h**) or eight (**i**,**j**) distinct experiments. Numbers in plots indicate the percentage of cells (**a**,**b**,**d**,**e**,**g**,**i**). Each symbol represents one individual mouse (**c**,**h**,**j**). ***: *P*<0.001, ****: *P*<0.0001 (unpaired *t*-test). Error bars indicate s.d.

To verify that the effects of Jmjd3 and Utx inactivation were cell-intrinsic, we generated mixed bone marrow chimeras, in which dKO and wild-type cells would compete in the same (wild type) environment. We transplanted a 1:1 mix of allelically marked dKO (CD45.2) and wild-type (CD45.1) haematopoietic progenitors into lethally irradiated wild-type (CD45.1/CD45.2) recipients. The dKO component showed accumulation of mature CD4 SP thymocytes and peripheral CD4^+^ lymphopenia, unlike wild-type competitors, indicating that the effect of Jmjd3 and Utx is cell-intrinsic (Fig. 2a). Accordingly, at all levels of chimerism, dKO cells were overrepresented relative to wild-type cells among mature CD4 SP thymocytes but underrepresented among CD4^+^ splenocytes (Fig. 2b,c). Control experiments with wild-type littermate donors showed that these effects were specific of dKO cells (Fig. 2b,c and Supplementary Fig. 4). These experiments demonstrate a cell-intrinsic need for Jmjd3 and Utx during the late stages of CD4^+^ T-cell development in the thymus and for the formation of normal CD4^+^ T-cell populations.

### Jmjd3 and Utx are needed for S1pr1 and Klf2 expression

The competitive disadvantage of dKO cells in mixed BM chimeras suggested that the effects of Jmjd3 and Utx disruption were partly masked in intact mice by shifts in the TCR repertoire of differentiating thymocytes. To examine this possibility, we fixed TCR specificity using the MHC II-restricted OT-II TCR transgene^30^. In H-2^b^ mice, the OT-II TCR promotes positive selection, resulting in the generation of large numbers of CD4 SP thymocytes and CD4^+^ T-cells expressing the transgenic Vα2 TCRα chain. Similar to their wild-type counterparts, dKO OT-II thymi had large populations of Vα2^hi^ cells, most of which were CD4 SP (Fig. 2d,e). In the Vα2^hi^ CD4^+^CD8^int^ subset, which comprises cells undergoing positive selection, surface expression of CD5 or CD69 proteins, both of which serve as indicators of TCR signalling, was similar in wild-type and dKO OT-II thymocytes (Fig. 2f). We conclude from these findings that Jmjd3 and Utx are not required for TCR signalling and positive selection *per se*.

**Figure 3:**
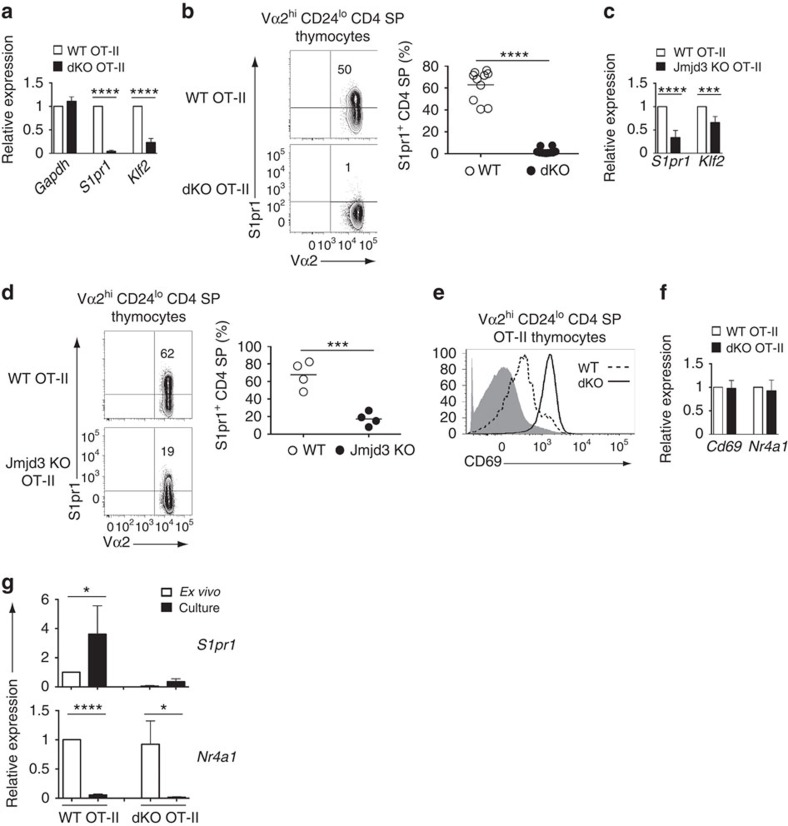
Jmjd3 and Utx are needed for *S1pr1* expression. (**a**) Quantitative RT-PCR showing expression of *Gapdh, S1pr1* and *Klf2* mRNA in OT-II transgenic wild-type (WT) and dKO mature thymocytes. Results are normalized relative to expression of the 18S rRNA gene and presented as fold change relative to wild type values (set as 1). Data is from five determinations from three independently sorted sample sets, except for *Gapdh* (two determinations from two sample sets). (**b**,**d**) Contour plots (left) of Vα2 and S1pr1 expression on gated Vα2^hi^ CD24^lo^ CD4 SP thymocytes from wild-type (WT) and dKO (**b**) or Jmjd3-KO (**d**) OT-II mice. Dot plots (right) shows frequency of S1pr1^hi^ cells among mature CD4 SP thymocytes; each symbol represents one individual mouse. Data comes from five (**b**) and four (**d**) distinct experiments. (**c**) RT-PCR analysis of *S1pr1* and *Klf2* mRNA expression in CD24^lo^ CD4 SP thymocytes from wild-type (WT) and Jmjd3-KO OT-II mice, expressed as in (**a**). Data is from three independent sample sets. (**e**) Overlaid histograms show expression of surface CD69 in Vα2^hi^CD24^lo^CD4 SP thymocytes from wild type (dotted line) and dKO (plain line) OT-II transgenic mice. Grey shaded histograms indicate expression in DP thymocytes from non-transgenic wild-type mice. Data is representative of four mice of each genotype, analysed in four distinct experiments. (**f**) RT-PCR analysis of *Cd69* and *Nr4a1* (encoding Nur77) expression in Vα2^hi^ CD24^lo^ CD4 SP thymocytes from indicated mice. Data is expressed as in (**a**) and comes from three independently sorted sample sets. (**g**) Bar graphs compare expression of *S1pr1 and Nr4a1* in sorted mature OT-II transgenic CD4 SP thymocytes either *ex vivo* or after cell suspension culture. Results are presented relative to WT *ex vivo* thymocytes (set as 1) after normalization on 18S rRNA expression. Data is from three independently sorted sample sets. *P<0.05, ***P<0.001 and ****P<0.0001, not significant in other cases (unpaired *t*-test). Error bars indicate s.d.

However, similar to mice with a diverse TCR repertoire, mature (Vα2^hi^ CD24^lo^) CD4 SP thymocytes were in markedly greater numbers in dKO than in wild-type OT-II mice (Fig. 2g,h). In addition, the number of peripheral dKO OT-II CD4^+^ T-cells was drastically reduced (Fig. 2g,h). The contrast between thymus and spleen prompted us to examine expression of S1pr1^31^,^32^, a sphingosine receptor needed for thymic egress. *S1pr1* mRNA expression was sharply reduced in mature OT-II dKO CD4 SP thymocytes (Fig. 3a) and the same was true of surface S1pr1 protein (Fig. 3b). Expression of *Klf2*, encoding a transcription factor important for late thymocyte maturation, including for *S1pr1* expression^33^, was also diminished (Fig. 3a). Single disruption of *Jmjd3* or *Utx* had partial effects on S1pr1 expression in mature OT-II thymocytes (Fig. 3c,d and Supplementary Fig. 5a), and caused a combination of thymocyte accumulation and lymphopenia, similar to but less marked than in dKO mice (Fig. 2i,j and Supplementary Fig. 5b,c).

Expression of *S1pr1* was impaired, with a trend towards reduced *Klf2* expression, in mature (TCR^hi^ CD24^lo^) dKO or Jmjd3-KO CD4 SP thymocytes carrying an endogenous TCR repertoire (Supplementary Fig. 6a,b) indicating that the impact of Jmjd3 and Utx on S1pr1 was not unique to the OT-II system. The same was true in mature (Vα2^hi^ CD24^lo^) CD8 SP thymocytes of mice carrying the MHC I-restricted P14 TCR transgene, which normally promotes positive selection of Vα2^hi^ CD8 SP thymocytes and T-cells (Supplementary Fig. 6c). Accordingly, P14-transgenic dKO mice had increased numbers of transgene-specific mature thymocytes and reduced numbers of CD8^+^ T-cells (Supplementary Fig. 6d–f).

We next examined how Jmjd3 and Utx disruption affected *S1pr1* expression. We first noted persistent expression of surface CD69 on mature Vα2^hi^ CD24^lo^ dKO OT-II CD4 SP thymocytes, but not on their wild-type counterparts which downregulated CD69 as they matured (Fig. 3e). Because surface CD69 is internalized upon association with S1pr1(ref. 34), it was possible that persistent CD69 surface expression on dKO cells resulted from reduced S1pr1 expression. Alternatively, it could reflect persistent high-level *Cd69* gene expression. Because *Cd69* is induced by TCR signalling, the latter hypothesis would suggest that mature dKO thymocytes had greater responsiveness to TCR engagements, which, in turn, would impair *S1pr1* expression. Indeed, TCR engagement repressed *S1pr1* in mature thymocytes (Supplementary Fig. 7), as previously shown in peripheral T-cells^31^, and the same was true of *Klf2* (Supplementary Fig. 7). To determine if disruption of Jmjd3 and Utx caused increased TCR signalling in mature thymocytes, we assessed Vα2^hi^ CD24^lo^ wild-type and dKO thymocytes for expression of the TCR-induced mRNAs encoding CD69 and the transcription factor Nur77 (ref. 35) (*Nr4a1*). Both were expressed at similar levels in wild-type and dKO cells (Fig. 3f), indicating that persistent expression of CD69 in mutant T-cells was post-transcriptionally controlled.

In addition, we reasoned that, if the reduced expression of *S1pr1* in mutant T-cells was caused by increased responsiveness to intrathymic signals, from TCR or other receptors, disrupting intrathymic interactions by placing mutant thymocytes in suspension culture should raise their *S1pr1* expression to wild-type levels. Validating this reasoning, expression of *S1pr1* in wild-type mature CD4 SP cells increased after *in vitro* culture, whereas that of *Nr4a1* decreased, attesting disrupted TCR signalling (Fig. 3g). However, expression of *S1pr1* in dKO mature cells was not significantly increased by *in vitro* culture, despite a reduction of their *Nr4a1* expression similar to wild-type thymocytes (Fig. 3g). We conclude from these experiments that Jmjd3 and Utx do not promote *S1pr1* expression by restraining thymocyte sensitivity to intrathymic signals.

### Jmjd3 and Utx control H3K27 methylation at S1pr1 and Klf2

**Figure 4:**
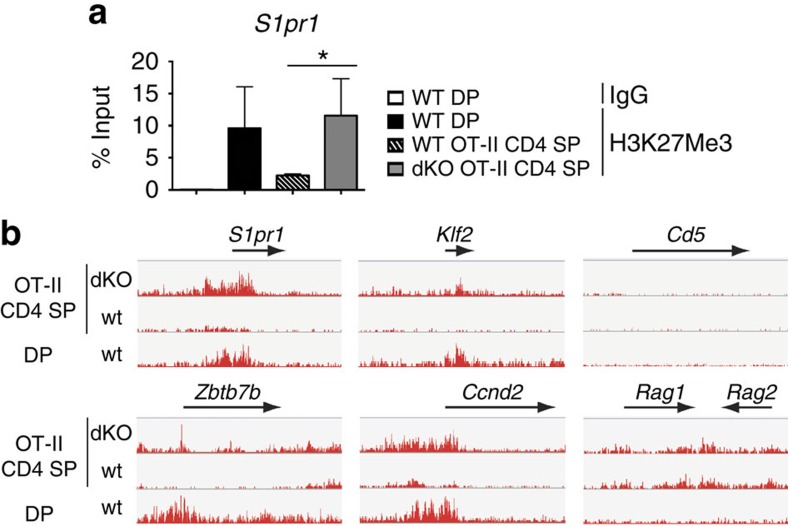
Jmjd3 and Utx redundantly promote H3K27Me3 demethylation. (**a**) H3K27Me3 amounts (mean±s.d.) at the *S1pr1* promoter were assessed by ChIP-qPCR in sorted DP thymocytes from wild-type mice carrying an endogenous TCR repertoire, or Vα2^hi^ CD24^lo^ CD4 SP thymocytes from wild type (WT) and dKO OT-II mice. H3K27Me3 amount values are shown as percentage of input. Data is from three independent experiments. *P<0.05 (unpaired *t*-test). (**b**) IGV browser tracks show the distribution (normalized sequence reads) of H3K27Me3 ChIPseq signals at indicated loci in wild-type DP and mature (Vα2^hi^ CD24^lo^) OT-II transgenic CD4 SP thymocytes.

To further investigate the mechanisms of reduced *S1pr1* expression, we performed H3K27Me3 chromatin immunoprecipitation (ChIP) at the *S1pr1* promoter. ChIP signals were higher in dKO than wild-type mature OT-II CD4 SP cells, indicating that Jmjd3 and Utx controlled H3K27 demethylation at *S1pr1* (Fig. 4a). To define the footprint of these enzymes on H3K27Me3 homeostasis and gene expression, we performed both ChIP followed by deep sequencing (ChIPseq) and microarray analyses on wild-type and dKO OT-II mature Vα2^hi^ CD24^lo^ CD4 SP thymocytes. ChIPseq confirmed the greater H3K27 trimethylation at *S1pr1* in dKO than in wild-type cells, and the same was true at *Klf2* (Fig. 4b). This indicated that reduced expression of both genes was associated with H3K27Me3 accumulation. In contrast, Jmjd3 and Utx disruption did not affect H3K27Me3 decoration at multiple other loci, including *Cd5* or *Rag1* and *Rag2*.

### Genome-wide impact of Jmjd3 and Utx

**Figure 5:**
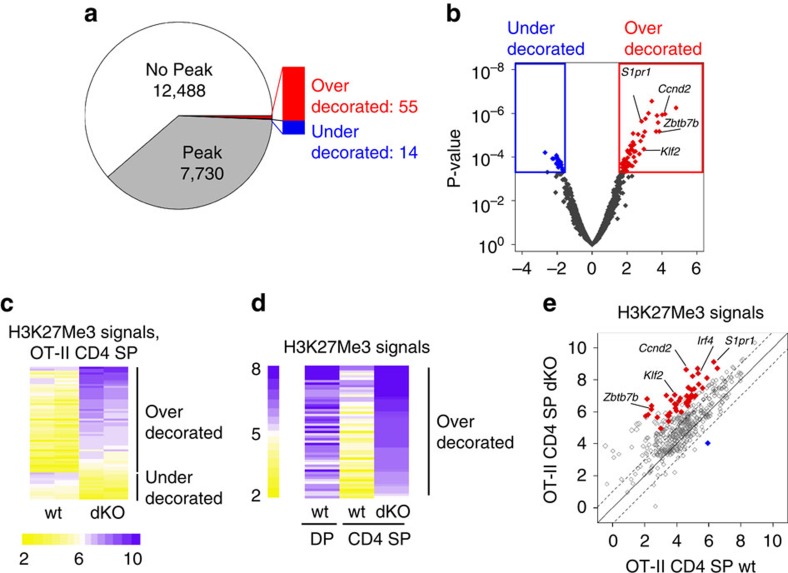
Genome-wide impact of Jmjd3 and Utx on H3K27 trimethylation in mature CD4 SP thymocytes. (**a**) Pie chart showing the number of gene promoters overlapping with H3K27Me3 peaks in wild-type or dKO OT-II mature CD4 SP thymocytes. The bar on the right highlights subsets of ‘Peak' genes with greater (overdecorated) or lower (underdecorated) H3K27Me3 signals in dKO than wild-type cells (defined in (**b**)) are indicated. (**b**) Volcano plot of ChIPseq data displaying for each locus the ratio of dKO over wild-type signals (*x* axis, log_2_ values) versus *P*-value (*y* axis, −log_10_ values). Coloured boxes and symbols define overdecorated (right, red) and underdecorated (left, blue) genes, defined by a twofold or greater change in H3K27Me3 signals with a 0.05 or lesser FDR. Relevant genes are indicated. (**c**) Heat map indicates ChIPseq signals (log_2_ values, colour-coding scale at bottom) on over- and underdecorated promoters (defined in (**b**)) in mature CD4 SP thymocytes from wild-type or dKO OT-II mice. Each lane represents a separate ChIPseq experiment and each row a separate gene promoter. Promoters are ranked by decreasing average signal values in dKO samples. (**d**) Heat map indicates ChIPseq signals (log_2_ values, colour-coding scale at left) in DP thymocytes (carrying an endogenous diverse repertoire, left column) and mature CD4 SP thymocytes from wild-type and dKO OT-II mice (right two columns, each showing the average of two independent samples). Promoters are ranked by decreasing average signal values in dKO samples (right lane). (**e**) Scatter plot show H3K27Me3 signals on a set of 449 promoters that normally undergo H3K27Me3 removal during the differentiation of DP to CD4 SP thymocytes. Each symbol depicts the signal (log_2_ value) at a given promoter in wild-type (*x* axis) versus dKO (*y* axis) mature CD4 SP OT-II thymocytes. Dotted lines indicate twofold changes. Filled red and blue symbols depict over- and underdecorated genes defined in (**b**).

We next assessed the 20218 unique genes included in ChIPseq and array analyses for ChIPseq signals (normalized read numbers) at promoters, defined as 4 kbp segments centred over a transcription start site. Using the peak-finding algorithm SICER, we found 7730 promoters overlapping an H3K27Me3 peak in either wild-type or dKO cells (Fig. 5a). At most of these 7730 ‘Peak' loci, there was little or no difference between wild-type and dKO H3K27Me3 signals (Fig. 5b and Supplementary Fig. 8a; examples in Supplementary Fig. 8b,c).

Among these 7730 ‘peak' loci, we found 55 ‘overdecorated' genes characterized by a 2-fold or greater H3K27Me3 signals in dKO than wild-type mature OT-II CD4 SP cells, accepting a 5% false discovery rate (FDR) (Fig. 5b,c and Supplementary Table 1; examples in Figs 4b and 5b and Supplementary Fig. 8d). Analyses of wild-type and dKO mature CD4 SP thymocytes expressing an endogenous TCR repertoire found increased H3K27Me3 signals in dKO cells at the vast majority of these 55 overdecorated loci, including *S1pr1* and *Klf2* (Supplementary Fig. 8d–f). Analyses in Jmjd3 or Utx single-deficient mature thymocytes suggested that Jmjd3 was primarily responsible for this effect (Supplementary Fig. 8d–f). We also identified 14 ‘underdecorated' genes, with paradoxically lower H3K27Me3 signals in dKO than in wild-type cells (Fig. 5b,c and Supplementary Fig. 8a). Since H3K27Me3 signals at most of these loci were low even in wild-type cells (Fig. 5c and Supplementary Fig. 8a), and there was no change in their expression (Supplementary Fig. 8g), we did not investigate them further.

Both *S1pr1* and *Klf2* promoters normally ‘clear' H3K27Me3 during the differentiation of DP into CD4 SP thymocytes (Fig. 4a,b), suggesting that the biological function of Jmjd3 and Utx could be to remove the mark at promoters induced during this developmental step. To evaluate this possibility, we performed H3K27Me3 Chipseq in wild-type CD69^lo^ DP thymocytes, the cell subset that contains precursors of positive selection that have not undergone TCR signalling. We used DP thymocytes carrying an endogenous TCR repertoire, as all OT-II transgenic thymocytes are subject to TCR engagement. At most of the 55 ‘overdecorated' promoters, wild-type H3K27Me3 signals were higher in DP than mature CD4 SP thymocytes (Fig. 5d). Reciprocally, we found that 449 genes normally undergo ‘clearance' of H3K27Me3 (defined as a 50% or greater drop in H3K27Me3 ChIPseq signals) during the differentiation of DP into CD4 SP thymocytes. Within that gene subset, average H3K27Me3 signals were significantly higher in dKO than wild-type mature CD4 SP thymocytes (*P*<2 × 10^−16^, paired *t*-test), with a twofold or greater increase in H3K27Me3 signal at 162 loci (36%) (Fig. 5e). We conclude from these findings that Jmjd3 and Utx are important for H3K27Me3 clearance during DP to CD4 SP thymocyte differentiation.

### H3K27Me3 accumulation impairs gene expression

**Figure 6:**
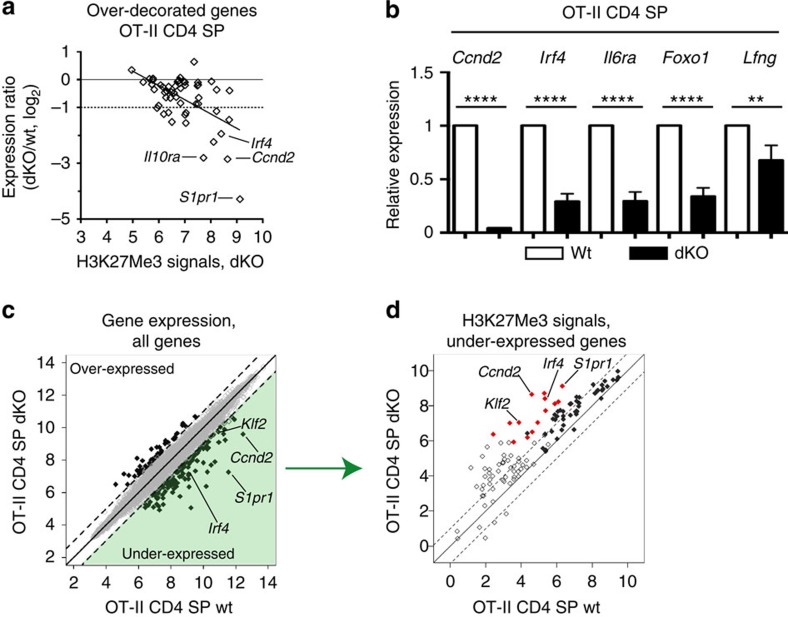
Impact of Jmjd3 and Utx on gene expression in mature CD4 SP thymocytes. (**a**) Scatter plot on the 55 over-decorated gene set, defined in Fig. 5b, shows H3K27Me3 signals in dKO OT-II CD4 SP thymocytes (log_2_ value, *x* axis) versus microarray expression ratios (dKO/WT OT-II CD4 SP thymocytes, log_2_ value, *y* axis). Horizontal lines indicate equal and 50%-reduced expression (plain and dotted lines, respectively). Each symbol represents one gene. The regression line (*r*=−0.54) is indicated. (**b**) Expression of indicated genes in mature (Vα2^hi^ CD24^lo^) CD4 SP thymocytes from wild-type (WT) and dKO OT-II transgenic mice is analysed by RT-PCR, and displayed as in Fig. 3a. Data is from three independent sample sets. **P<0.01 and ****P<10^−4^ (unpaired *t*-test). Error bars indicate s.d. (**c**) Scatter plot shows microarray gene expression (log_2_ values, full 20218 gene set) in wild-type (*x* axis) versus dKO (*y* axis) mature CD4 SP OT-II thymocytes. Dotted lines indicate twofold changes. Thick symbols depict genes with a significant (FDR<0.05) twofold or greater change in expression. The green-shaded area depicts the 115 under-expressed genes analysed in Fig. 5d. (**d**) Scatter plot show H3K27Me3 signals (log_2_ values) in wild-type (*x* axis) versus dKO (*y* axis) mature CD4 SP OT-II thymocytes on 115 genes underexpressed in dKO compared with wild-type cells (green-shaded area in (**c**)). Dotted lines indicate twofold changes. Filled red and blue symbols depict over- and underdecorated genes defined in Fig. 5b.

Microarray analyses showed a significant (*P*<0.05) reduction in expression at 40 of the 55 overdecorated genes in dKO mature thymocytes, and the reduction was >50% at 14 of them, including *S1pr1* and *Klf2* (Fig. 6a, and Supplementary Table 1). Although the correlation was not perfect, the effect on gene expression was significantly correlated with H3K27Me3 signals in dKO cells (Fig. 6a, *P*<10^−4^, Pearson's *r*=−0.54). The greater impact on genes with high H3K27Me3 signals presumably reflected that low H3K27Me3 signals in that overdecorated gene subset indicate methylation changes in a minor fraction of cells. RT-PCR analyses confirmed microarrays findings at *Ccnd2*, *Irf4*, *Il6ra*, *Foxo1* and *Lfng* (Fig. 6b), in addition to *S1pr1* and *Klf2* (Fig. 3a). Of note, *Zbtb7b* expression was not reduced in dKO cells (Supplementary Table 1), consistent with the presence of mature CD4 SP thymocytes. Accordingly, H3K27Me3 decoration at the *Zbtb7b* promoter remaining substantially lower in dKO CD4 SP than in DP thymocytes (Fig. 4b and Supplementary Table 1). We conclude from these analyses that Kdm6-family demethylases are needed for H3K27Me3 homeostasis and expression of a limited gene subset in CD4 SP thymocytes.

Because Jmjd3 and Utx have functions independent from their demethylase activities^14^,^16^,^17^,^18^,^36^,^37^, we examined if all changes in gene expression in dKO cells were associated with changes in H3K27Me3 signals. On the full 20218-gene set, microarray analyses identified 115 ‘underexpressed' genes, with a 50% or greater drop in expression in dKO cells (with FDR<0.05) (Fig. 6c and Supplementary Table 2). Among these, most (70) had promoters overlapping with an H3K27Me3 peak, of which 68 had greater H3K27Me3 signals in dKO than in wild-type thymocytes (Fig. 6d, filled symbols). This reinforces the concept that effects of Jmjd3 and Utx on gene expression were mediated at least in part by their demethylase activity.

However, the additional 45 genes underexpressed in dKO cells did not overlap with H3K27Me3 peaks (Fig. 6d, open symbols), including *Ccr7* and *Sell* (encoding CD62L), which were previously shown to be Klf2-dependent^33^,^38^, and *Ccr4* (Supplementary Fig. 9a). Conversely, using the same FDR stringency (<0.05), 41 ‘overexpressed' genes had at least twofold greater expression in dKO than wild-type cells, although the change was typically modest (Fig. 6c). Athough a few of these overlapped with an H3K27Me3 peak, increased expression was not associated with changes in H3K27Me3 decoration (Supplementary Fig. 9b). These findings are consistent with the idea that Jmjd3 or Utx can affect gene expression indirectly, or through demethylase-independent activities.

### Demethylase-dead Uty fails to rescue S1pr1 expression

**Figure 7:**
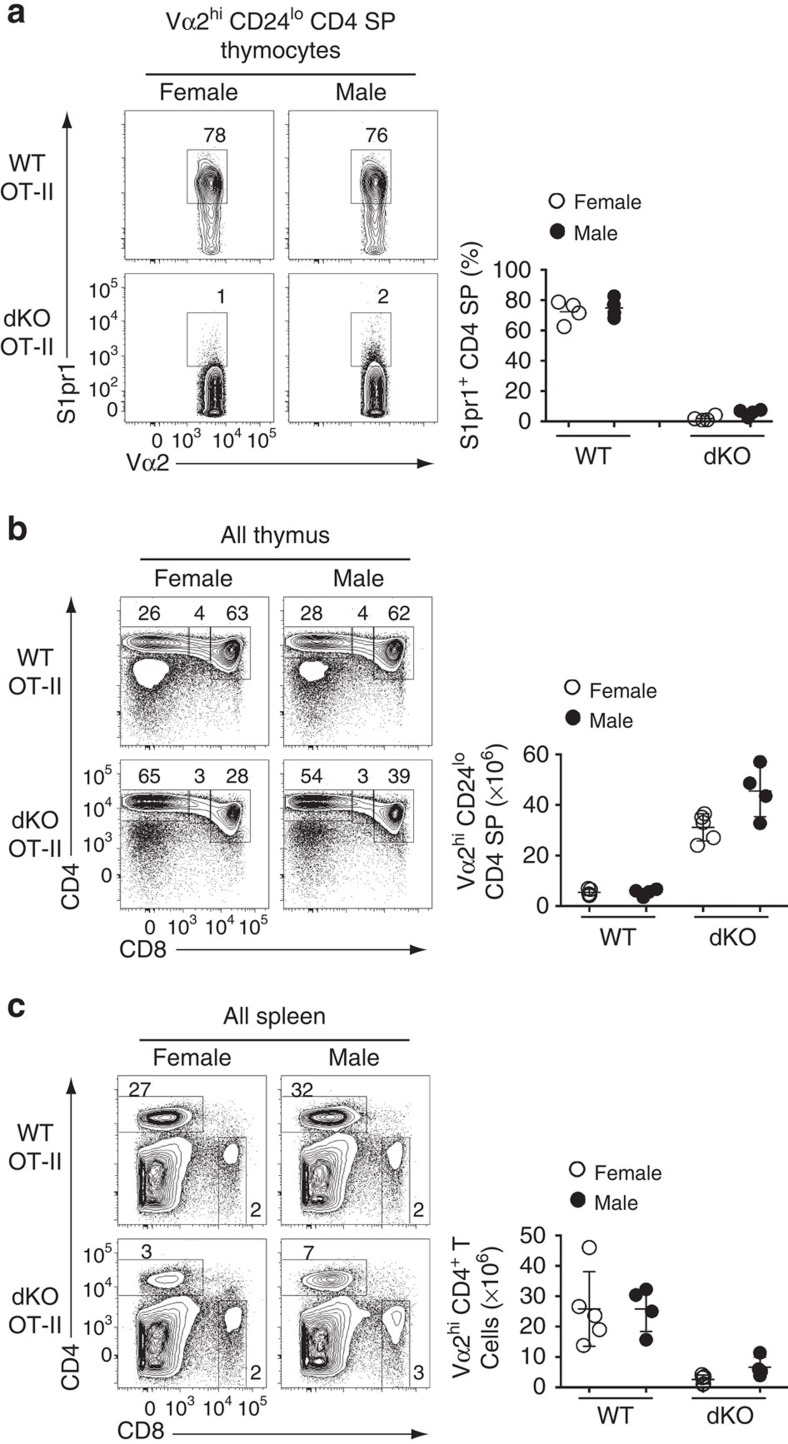
Demethylase-inactive Uty fails to support *S1pr1* expression. (**a**) Contour plots (left) show expression of S1pr1 and Vα2 on mature thymocytes from wild-type (WT) and dKO OT-II transgenic female or male mice. Dot plots (right) show the frequency of S1pr1^hi^ cells among mature CD4 SP thymocytes. Each symbol represents one individual mouse. (**b**,**c**) Contour plots (lefts) show CD4 and CD8 expression on thymocytes (**b**) or splenocytes (**c**) from mice analysed in (**a**). Dot plots (right) show absolute numbers of mature Vα2^hi^ CD4 SP thymocytes (**b**) or CD4^+^ T-splenocytes (**c**). Each symbol represents one individual mouse. Results are representative of four mice of each gender and genotype analysed in four distinct experiments (**a**–**c**). *: *P*<0.05 (unpaired two-tailed *t*-test). Error bars indicate s.d.

The evidence for demethylase-independent effects of Jmjd3 and Utx prompted us to investigate further whether their effect on *S1pr1* was mediated by H3K27Me3 accumulation. To this end, we took advantage from the fact that the Y-chromosome homolog of *Utx*, *Uty*, encodes a protein with little if any demethylase activity, yet carries demethylase-independent functions of Utx^11^,^14^,^16^,^17^,^18^,^36^. Thus, if demethylase-independent activities of Utx were sufficient to promote S1pr1 expression, Uty should promote S1pr1 expression in male mice lacking Jmjd3 and Utx. This was not the case, as expression of S1pr1 was similarly low in OT-II dKO mice of both genders (Fig. 7a). Furthermore, Uty minimally increased the numbers of CD4 SP thymocytes and spleen CD4^+^ T-cells (Fig. 7b,c), the latter remaining well below that in OT-II Jmjd3-deficient mice (6.6±1.6 × 10^6^ versus 15.41±1.7 × 10^6^, mean±s.e.m., Figs 2j and 7c). This supports the conclusion that H3K27Me3 demethylation at *S1pr1* is needed for its expression.

### Enforced S1pr1 expression prevents thymocyte accumulation

**Figure 8:**
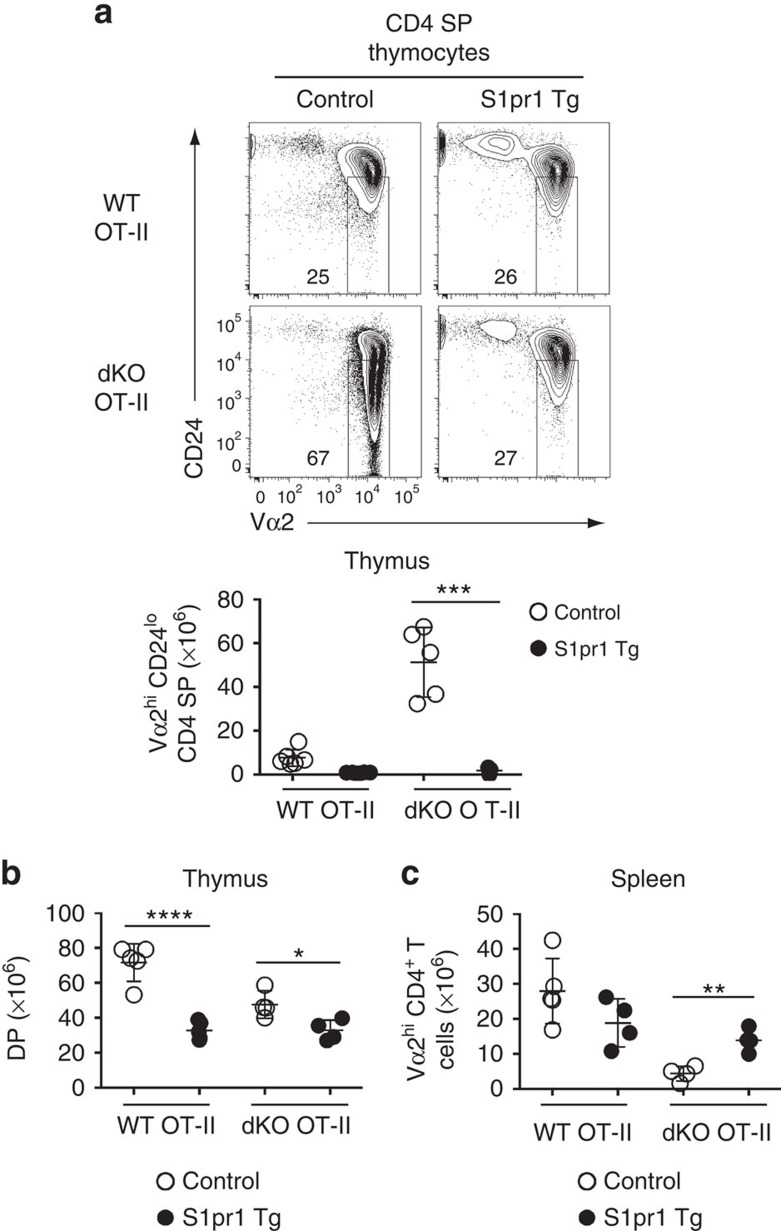
Enforced S1pr1 expression rescues egress of dKO thymocytes. (**a**) (top) Contour plots of CD24 and Vα2 expression on CD4 SP thymocytes from wild-type (WT) and dKO OT-II mice carrying (S1pr1 Tg) or not (Control) the *S1pr1* transgene. Numbers in plot indicate the percentage of cells in mature CD24^lo^ Vα2^hi^ gate. Dot plot (bottom) shows absolute numbers of mature CD4 SP thymocytes from indicated mice. (**b**,**c**) Absolute numbers of DP thymocytes (**b**) and spleen Vα2^hi^ CD4^+^ T-cells (**c**) from the mice shown in (**a**). (**a**–**c**) Statistical significance was determined by unpaired *t*-test. *: *P*<0.05, **: *P*<10^−2^,***: *P*<10^−3^, ****: *P*<10^−4^. Each symbol on dot plots represents one individual mouse and results are representative of or combined from four independent experiments. Error bars indicate s.d.

The requirement for Jmjd3 and Utx for expression of *Klf2* and chemokine receptors involved in intrathymic migration, including CCR4 and CCR7, raised the possibility that the accumulation of dKO thymocytes was not caused by impaired S1pr1 expression. To address this possibility, we enforced *S1pr1* expression in OT-II thymocytes lacking both Jmjd3 and Utx. Expression of an *S1pr1* transgene resulted in a marked drop in Vα2^hi^ CD24^lo^ CD4 SP thymocytes (Fig. 8a), whereas it had a much more modest effect on the DP subset (Fig. 8b). Furthermore, expression of the transgene increased the numbers of Vα2^hi^ spleen CD4^+^ T-cells (Fig. 8c). We conclude from these experiments that impaired *S1pr1* expression is a key factor limiting the egress of dKO thymocytes.

In summary, we demonstrate that Jmjd3 and Utx demethylases are redundantly required for proper T-cell development. We further show that these molecules promote the normal developmental clearance of H3K27Me3 at and expression of specific genes, including *S1pr1*.

## Discussion

The present study demonstrates that Jmjd3 and Utx H3K27Me3 demethylases are redundantly required for the terminal steps of T-cell development, and notably for expression of *S1pr1*, a gene needed for thymic egress. As a result, Jmjd3 and Utx are needed for the generation of a normal T-cell repertoire. In the immune system, genetic analyses had shown that Jmjd3 contributes to macrophage and T-cell effector differentiation^28^,^39^, and to the differentiation of invariant natural killer T-cells^40^, a population of innate-like lymphocytes recognizing lipid antigens bound to CD1d molecules^41^. However, the full impact of Jmjd3 and Utx on gene expression and cell differentiation has remained unclear, as none of these reports had assessed cells lacking both enzymes. The present study demonstrates essential and partly overlapping functions of Jmjd3 and Utx in developing T-cells.

Previous studies have highlighted the importance of Jmjd3 and Utx, the two molecules with H3K27Me3 demethylase activity, in development and disease. These molecules are involved at multiple steps of mouse embryogenesis and during cell differentiation^14^,^15^,^16^,^17^,^18^,^19^,^28^,^36^,^39^. In addition, UTX mutations were found in a subset of children with Kabuki syndrome, a congenital disorder associating multiple developmental defects^42^. Furthermore, Jmjd3 and Utx serve opposite functions during T-cell leukemogenesis, the former promoting tumorogenesis whereas the latter acts as a tumour suppressor gene^43^,^44^.

While Jmjd3 and Utx both have catalytic demethylase activities, the contribution of such activity to their biological functions has remained elusive. Most mechanistic studies addressing this question have focused on early embryonic differentiation, in which Utx is the key physiological player^14^,^16^,^17^,^18^,^36^. Utx is notably required for mesoderm formation and expression of the *T* gene, encoding the key mesoderm inducer Brachyury^17^. Utx molecules bind the *T* promoter *in vivo*, suggesting a direct effect of Utx on *T* transcription. However, neither mesoderm formation nor *T* expression require Utx demethylase activity: both are rescued by Uty, which lacks demethylase activity, or by a Utx mutant lacking demethylase activity^14^,^16^,^17^,^18^,^36^. Strikingly, Uty supports the development of embryos harbouring disruptions of both *Utx* and *Jmjd3*, questioning the importance of Utx and Jmjd3 demethylase activity for cell differentiation^21^. Although it has been reported that a demethylase-inactive mutant of Jmjd3 fails to rescue the postnatal death of Jmjd3-deficient mice^15^, that report did not document expression of the mutant Jmjd3 protein.

Contrasting with *T* expression and early embryonic development, our study supports the concept that the catalytic activity of Jmjd3 and Utx is crucial for their impact on gene expression and cell differentiation, by showing that *Uty* does not support expression of *S1pr1* in male thymocytes lacking both *Jmjd3* and *Utx*. Of note, similar to the broad distribution of *Utx* and *Uty* in developing embryos (with limited tissue specificity in the expression of either gene)^16^,^20^, data from the Immgen data base indicates widespread expression of both genes in immune cells, including throughout T-cell development (www.immgen.org and Mingueneau *et al*.^45^). Thus, although the generation of conditional alleles encoding catalytically inactive versions of Jmjd3 and Utx will be needed to formally demonstrate that H3K27Me3 demethylase activity is required for expression of *S1pr1* in mature thymocytes, our results support such a conclusion, and the broader notion that the full impact of Jmjd3 and Utx on transcription requires their demethylase activity.

In non-proliferating but actively differentiating thymocytes, in which H3K27Me3 cannot be diluted during cell division, the footprint of Jmjd3 and Utx on H3K27Me3 homeostasis is highly specific. Even though a 5% FDR cut-off presumably eliminates authentic functional targets, we estimate that the combined activity of Jmjd3 and Utx is required for H3K27Me3 homeostasis at less than 1% of gene promoters. This suggests that the activity of H3K27 methylases Ezh1 and Ezh2 is tightly controlled and acts as the rate-limiting factor for the propagation of H3K27Me3 in the genome. Most of the effect on H3K27Me3 in mature thymocytes appears attributable to Jmjd3, as disruption of Utx *per se* only minimally affects promoter decoration.

Although Jmjd3 or Utx ChIP attempts were not successful with our current antibodies, it was reported that Jmjd3 binds the *S1pr1* promoter in the HL60 cell line^46^, supporting the idea of a direct Jmjd3 involvement. Of note, that study documented Jmjd3 binding to more than 8,000 genes, of which only a minor subset (fewer than 200) depended on Jmjd3 for their expression^46^. Conversely, Utx binds the *T* promoter, yet promotes its activity through demethylase-independent mechanisms^17^. Thus, for both Jmjd3 and Utx, promoter recruitment does not appear as a proper predictor of involvement in H3K27Me3 removal.

Although we found a clear link between H3K27Me3 retention and reduced gene expression in cells both lacking Jmjd3 and Utx, the correlation was not absolute. In fact, a perfect correlation between the impact of these enzymes on H3K27Me3 decoration and transcription was not expected, for two complementary set of reasons. First, Jmjd3 and Utx can promote gene-specific transcription without a corresponding impact on local H3K27Me3 decoration. Indeed, we found that these enzymes affected expression of genes with no H3K27Me3 decoration. Such effects presumably involve two non-mutually exclusive mechanisms. Jmjd3 and Utx can affect locus-specific transcription indirectly, through their impact on the expression of *trans*-acting transcription factors, or directly, through demethylase-independent mechanisms. Illustrating the first possibility, Jmjd3 and Utx control of *Klf2* expression may account for their impact on Klf2 targets *Ccr7* and *Sell* (encoding CD62L). Direct, demethylase-independent effects have been identified in multiple contexts for both Jmjd3 and Utx. In addition to those of Utx in ES cells, such functions of Jmjd3 promote transcriptional activation by T-bet^37^, a transcription factor required for the differentiation of Th1 effector CD4^+^ T-cells^47^. Multiple mechanisms appear to support such functions. Jmjd3 links T-bet to chromatin remodelling complexes at a specific subset of T-bet target genes, whereas both Jmjd3 and Utx have been reported to associate with the MLL enzymatic complexes involved in H3K4 methylation and promoting gene expression^10^,^13^,^43^,^48^,^49^.

Conversely, Jmjd3 and Utx-dependent clearance of H3K27Me3 was not strictly required for gene expression. This points to the existence of additional levels of control on transcriptional repression mechanisms downstream of H3K27Me3. H3K27Me3-mediated gene repression is thought to primarily involve H3K27Me3-bound PRC1 complexes, including H3K27Me3-binding subunits (Cbx proteins) and components responsible for transcription repression. Despite the similarity among isoforms of Cbx proteins, recent studies have highlighted unique functional and biochemical properties specific of each variant^8^,^50^,^51^. Similarly, there is much plasticity in the association of silencing-mediating subunits to PRC1 (ref. 8). In addition, Polycomb-independent mechanisms have been proposed to contribute to the repressive effects of H3 K27 trimethylation^8^, including interference with the ‘activating' acetylation of H3 K27 and effects on nucleosome packaging or DNA opening for transcriptional elongation^46^,^52^. Our findings suggest that such ‘effector' mechanisms of H3K27Me3-mediated transcriptional repression are subject to control, possibly by extracellular signals.

Our study also documents that Jmjd3 and Utx are not absolutely required for gene-specific clearance of H3K27Me3 during the differentiation of DP into CD4 SP thymocytes. Surprisingly, H3K27Me3 removal at *Zbtb7b*, encoding the CD4-differentiating factor Thpok, was only modestly affected by Jmjd3 and Utx disruption; accordingly, neither enzyme was needed for *Zbtb7b* expression and for the differentiation of CD4^+^-lineage thymocytes. Such observations in non-dividing thymocytes imply the existence of Jmjd3- and Utx-independent H3K27Me3 removal mechanisms. These may involve some degree of promiscuity in substrate specificity among JmjC-domain demethylases, as recently suggested^53^. Alternatively, it is possible that demethylase-independent mechanisms contribute to remove H3K27Me3 at select genes. One particularly interesting possibility is the transcription-associated substitution of conventional histone H3 by variant H3.3 molecules^54^; this mechanism could conceivably replace methylated histone H3 by newly synthesized variant molecules that had not been subject to K27 methylation.

Many of the genes dependent on Jmjd3 and Utx for H3K27Me3 clearance and expression, including *S1pr1*, *Klf2*, *Ccnd2*, *Il6ra*, *Foxo1* or *Lfng*, are characteristic of mature T-cells and induced during late thymocyte differentiation. Although it is possible that the impact of Jmjd3 and Utx disruption on *S1pr1* is amplified by a ‘sampling bias', as cells that fail to express *S1pr1* accumulate in the thymus, this would not explain the impact on other genes. The preferential impact of Jmjd3 and Utx on such ‘late maturation' genes is consistent with a role for these enzymes in integrating extracellular cues at promoters activated during terminal thymocyte differentiation. The demethylase activity of these enzymes requires oxygen and α-ketoglutarate, produced from glucose or glutamine^55^, which control their function in embryonic stem cells. Thus, it is possible that Jmjd3 and Utx activation, and action at *S1pr1*, serves as a postselection licensing signal for thymocytes having undergone positive selection and increased their metabolic activity.

In summary, we demonstrate extensive H3K27Me3 changes during the differentiation of CD4^+^ T-cells from DP thymocytes and show that enzymatic removal of H3K27Me3 is required for the proper outcome of this differentiation step. Our unique approach, in non-proliferating cells, demonstrates an *in vivo* requirement for H3K27Me3 demethylase activity in gene expression during cell differentiation.

## Methods

### Generation of conditional Jmjd3- or Utx-deficient mice

The targeting vector for each conditional knockout mouse included an inserted neomycin (Neo) cassette flanked by Frt, and three loxP sites flanking the Neo cassette and *Jmjd3* exons 14–20 or *Utx* exon 24 (covering exon(s) encoding the JmjC domain). After transfection of the targeting vector into embryonic stem (ES) cells, recombinant ES cells were selected by G418 and FIAU (1–2'-deoxy-2'-fluoro-beta-D-arabinofuranosyl-5-iodouracil), screened by Southern blot analyses, and injected into blastocysts derived from C57BL/6 mice. Deletion of Frt-flanked Neo cassette was achieved by crossing of mice heterozygous for the recombinant allele to mice expressing Flpe recombinase driven by β-actin promoter, resulting in the floxed allele (*Jmjd3*^*f*^ or *Utx*^*f*^). Similarly, the deleted allele (*Jmjd3*^*−*^ or *Utx*^−^) was obtained by Cre-mediated deletion of the floxed sequence through mating of mice. The genotype of recombinant mice was verified by Southern blot analysis for the first two generations and subsequently determined by PCR analysis. We used Cre-negative *Jmjd3*^f/f^ or *Utx*^f/f^ littermates as ‘wild-type' controls in this study. For the generation of OT-II or P14-transgenic mutant mice or *S1pr1* transgenic mice, Jmjd3 and Utx mutant lines were crossed to OT-II^30^ or P14 (ref. 56) TCR transgenic, or *S1pr1* (ref. 57) transgenic mice. Primers for genotyping are listed in Supplementary Methods.

### Mice

CD45.1 and CD45.2 C57BL/6 mice were obtained from the National Cancer Institute Animal Production Facility. All transgenic mice were maintained heterozygous for the transgene. Experiments were performed on female mice except when otherwise indicated. Mice were housed in specific pathogen-free facilities and analysed between 6 and 16 weeks of age. Animal procedures were approved by the NCI Animal Care and Use Committee.

### Antibodies

The following antibodies were purchased from either BD Biosciences or eBioscience (clone name and final concentration used in parenthesis): CD4 (RM4.5, 1 μg ml^−1^), CD8α (53-6.7, 2.5 μg ml^−1^), CD8β (53-5.8, 2.5 μg ml^−1^), TCR (H57–597, 1 μg ml^−1^), CD24 (M1/69, 2.5 μg ml^−1^), CD69 (H1.2F3, 1 μg ml^−1^), CD44 (IM7, 1 μg ml^−1^), CD45.2 (104, 0.5 μg ml^−1^), anti-CD45.1 (A20, 1 μg ml^−1^), CD5 (53-7.3, 1 μg ml^−1^), MHC II I-A/I-E (M5/114.15.2, 1 μg ml^−1^) and Vα2 (B20.1, 1 μg ml^−1^). The rat anti-mouse S1pr1 antibody was kindly provided by Dr Jason Cyster (University of California, San Francisco) and subsequently purchased from R&D Systems (MAB7089). The Jmjd3 antibody for immunoblot analysis was generated in rabbits using a peptide (amino acids 211–230 of mouse Jmjd3) as an epitope, and affinity-purified; the Utx antibody was purchased from Bethyl laboratories (A302–374A). Final concentrations for Jmjd3 and Utx antibodies were 2 and 0.2 μg ml^−1^ for immunoprecipitation and immunoblotting, respectively. Anti-H3K27Me3 (#17–622) antibodies for ChIPseq were purchased from Millipore.

### Cell preparation and flow cytometry

Thymocytes and splenocytes were isolated from mice (6–10 weeks of age) and analysed by immunostaining and flow cytometry as described^58^. Data were acquired on a LSR Fortessa cytometer (BD Biosciences) and analysed with Flowjo software. The S1pr1 staining was performed by incubating with the rat anti-mouse S1pr1 antibody (2 μg/10^6^ cells), followed by staining with anti-rat IgG PE (eBioscience, 12-4822-82, 10 μg ml^−1^). Cells were then washed and blocked with 2% normal rat serum before being incubated with additional antibodies. For sorting of mature CD4 SP thymocytes (TCR^hi^ CD24^lo^), CD4 SP thymocytes were pre-enriched by using a Dynal mouse CD4 negative isolation kit (Invitrogen), stained with antibodies, and sorted on a BD FACSAria cell sorting system (BD Biosciences).

### Bone marrow chimeras

T-cell-depleted (Pan T Dynal kit, Invitrogen) bone marrow was isolated from CD45 disparate animals, mixed at a 1:1 ratio, and injected into lethally irradiated (900 rads) recipients heterozygous for CD45.1 and CD45.2, which were analysed 8 weeks after transplantation.

### *In vitro* thymocyte culture

Sorted mature thymocytes were resuspended in RPMI-1640 medium (supplemented with 10% FCS, 1% Pen/Strep/Glu) and incubated overnight in presence or absence of plate bound anti-CD3 (145-2C11; BD Biosciences, coated with 1 μg ml^−1^ solution) at 37 °C, 5% CO_2_.

### Microarray and quantitative RT-PCR analyses

Total RNA from sorted Vα2^hi^ CD24^lo^ (OT-II) or TCR^hi^ CD24^lo^ (endogenous repertoire) CD4 SP thymocytes or cultured thymocytes was isolated using RNeasy Plus Mini Kit (Qiagen). Microarray analyses (Affymetrix Mouse Gene 1.0 ST array) were performed by the NCI microarray facility following the manufacturer's recommendation (Affymetrix). For quantitative RT-PCR analysis, cDNA was synthesized from total RNA using ThermoScript RT-PCR system (Invitrogen), and the PCR was performed on a 7500 Real time PCR system (Applied Biosciences). The mRNA levels of target genes were normalized with 18S rRNA, and represented as the fold change over wild-type (or unstimulated control, Supplementary Fig. 7) values (set arbitrarily as 1). Primer sequences will be provided upon request. Microarray data were analysed using Partek Genomic Suite from Affymetrix ‘cel' files generated at the NCI microarray facility. Primers for RT-PCR are listed in Supplementary Methods.

### ChIP

Sorted mature (TCR^hi^ or Vα2^hi^ CD24^lo^) thymocytes were digested with Mnase (Sigma-Aldrich, N3755, 0.4 U ml^−1^), followed by sonication to generate mainly mononucleosomes with minor fraction of dinucleosomes. Sonicated supernatants were pre-cleared for 1 h with protein G beads at room temperature and then immunoprecipitated with anti-H3K27Me3 (10 μg ml^−1^, pre-adsorbed on Protein G beads) or control mouse monoclonal IgG (10 μg ml^−1^) overnight. Immunoprecipitated complexes were collected on protein G beads and DNA was purified by PCR Purification Kit (Qiagen) and analysed by quantitative (Sybr green) PCR (see Supplementary Methods for primer sequence). Data is presented as a percent input of each IP sample relative to input chromatin.

### ChIPseq and data analysis

The fastq files were aligned against the genome reference mm9 and promoter reads (±2 kb from the transcription start site) detected for each gene using the Rsubread package in R^59^. Promoter counts were normalized by loess using csaw package^60^. Broad peaks were detected using SICER^61^, and promoter reads overlapping with SICER peaks in two biological replicates per group (dKO and WT) further analysed for differential binding using the glmQLFit function in EdgeR^62^,^63^. Offsets were used to generate normalized bedgraph files for visualization in IGV. Entries redundant for gene name and boundaries were eliminated, leaving a total of 20218 unique entries processed for combined ChIPseq-microarray analyses.

### Statistical analyses

Statistical analyses were performed using Prism software except as noted above for microarrays and ChIPseq data. Bars in graphs indicate average±s.d. Except where otherwise indicated, comparisons were performed by two-tailed unpaired Student's *t*-test. Two-tailed paired *t*-test was used where biologically appropriate (for example, competitive bone marrow chimeras). Significance levels (*P*-values) are indicated on figures. For statistical comparisons, sample size was >3 and was determined empirically based on pilot analyses. We used neither randomization nor blinding; animals were excluded from analyses only on the basis of criteria (for example, age or poor health status) unrelated to the experiment result.

## Additional information

**How to cite this article:** Manna, S. *et al*. Histone H3 Lysine 27 demethylases Jmjd3 and Utx are required for T-cell differentiation. *Nat. Commun*. 6:8152 doi: 10.1038/ncomms9152 (2015).

## Supplementary Material

Supplementary Information Supplementary Figures 1-9, Supplementary Tables 1-2 and Supplementary Methods
