# Supplementary material for: Histone H3 Lysine 27 demethylases Jmjd3 and Utx are required for T-cell differentiation
Source: Nat Commun. 2015 Sep 2;6:8152. doi: 10.1038/ncomms9152 (PMC4569738; doi:10.1038/ncomms9152)
Supplement: Supplementary Information — Supplementary Figures 1-9, Supplementary Tables 1-2 and Supplementary Methods [file ncomms9152-s1.pdf]

Supplementary Figures

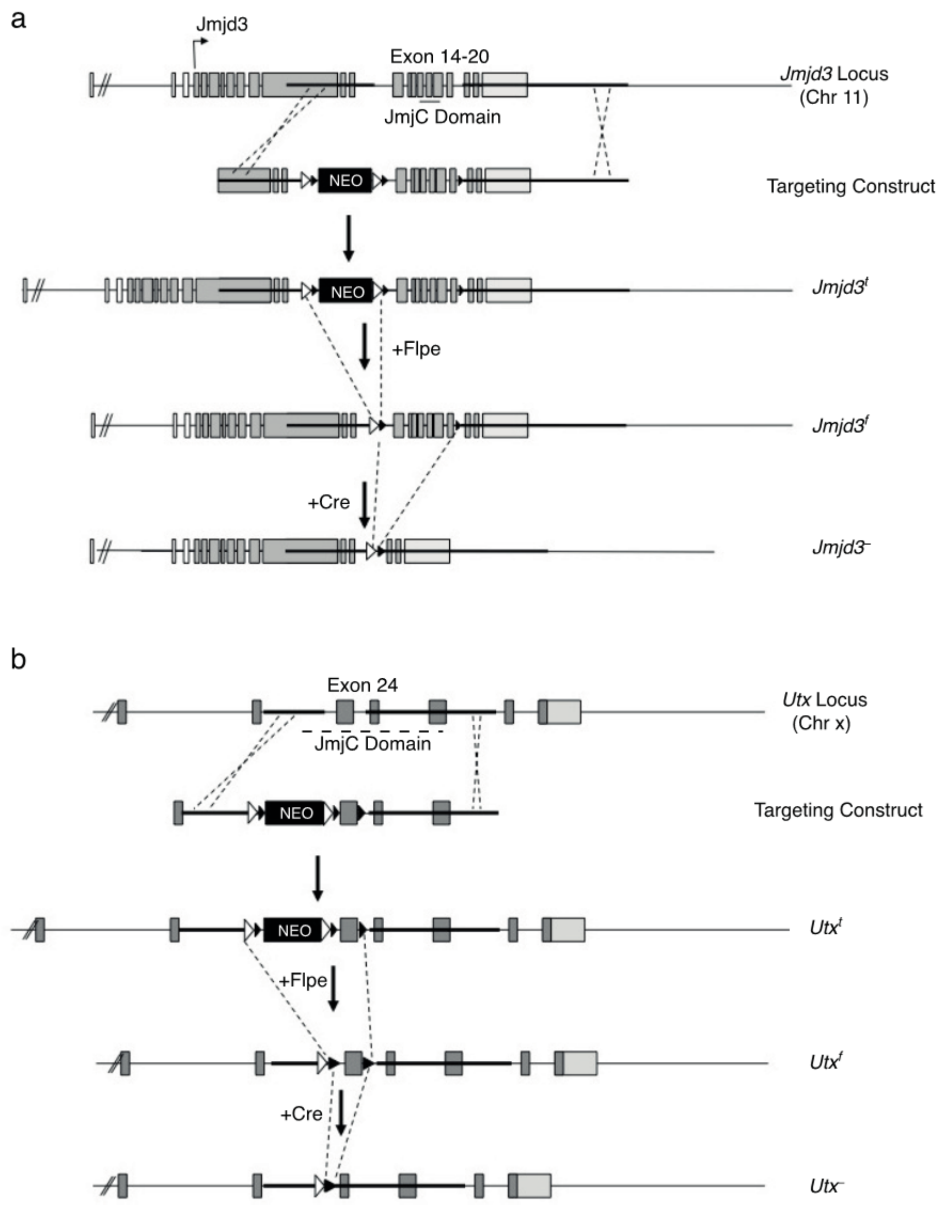

Supplementary Figure 1

Targeting strategy for the generation of conditional Jmjd3- or Utx conditional mice.

(**a, b**) Structure of wild-type *Jmjd3* (**a**) or *Utx* (**b**) loci and targeting vectors. The targeting vector for each gene contains an inserted neomycin (Neo) cassette flanked by Frt (white arrowheads), and three LoxP sites (black arrowheads) flanking the Neo cassette and exon(s) encoding the catalytic JmjC domain for each enzyme. Homologous recombination in embryonic stem cells results in the targeted allele ($Jmjd3^{t}$ or Utx^{t}).

Subsequent deletion of Frt-flanked Neo cassette by Flpe recombinase gives rise to the floxed allele (*Jmjd3*^{f} or *Utx*^{f}), which can be further modified to the deleted allele (*Jmjd3*$^{-}$ or *Utx*$^{-}$ by the Cre recombinase.

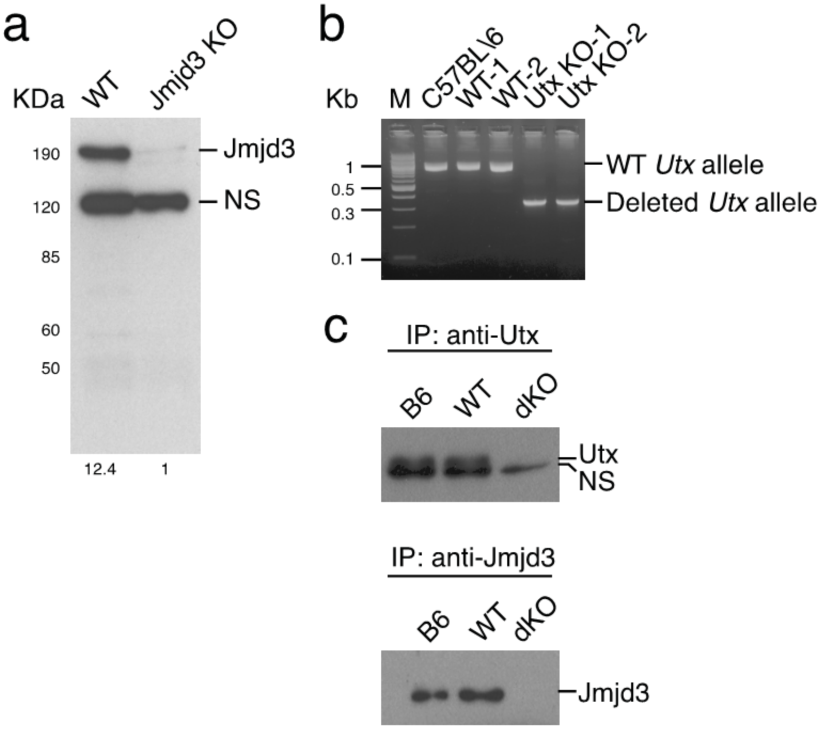

Supplementary Figure 2

Deletion of Jmjd3 and Utx alleles.

(**a**) Depletion of Jmjd3 protein in *Jmjd3*$^{f/f}$ *Cd4*-Cre (*Jmjd3* KO) thymocytes as determined by immunoprecipitation (IP) and immunoblot analyses with a Jmjd3 antibody. Numbers below the image are the relative intensity ratio of the Jmjd3 bands measured by densitometric analyses. NS: non-specific band.

(**b**) Deletion of the floxed region in *Utx* allele as monitored by genomic PCR. Two biological replicates are shown for wild-type (WT) and *Utx* $^{f/f}$ *Cd4*-Cre mice (*Utx* KO), using genomic DNA purified from total thymocytes of each mouse.

(**c**) Depletion of both Jmjd3 and Utx proteins in double-deficient (dKO) thymocytes as determined by IP/immunoblot analyses with a Jmjd3 or Utx antibody.

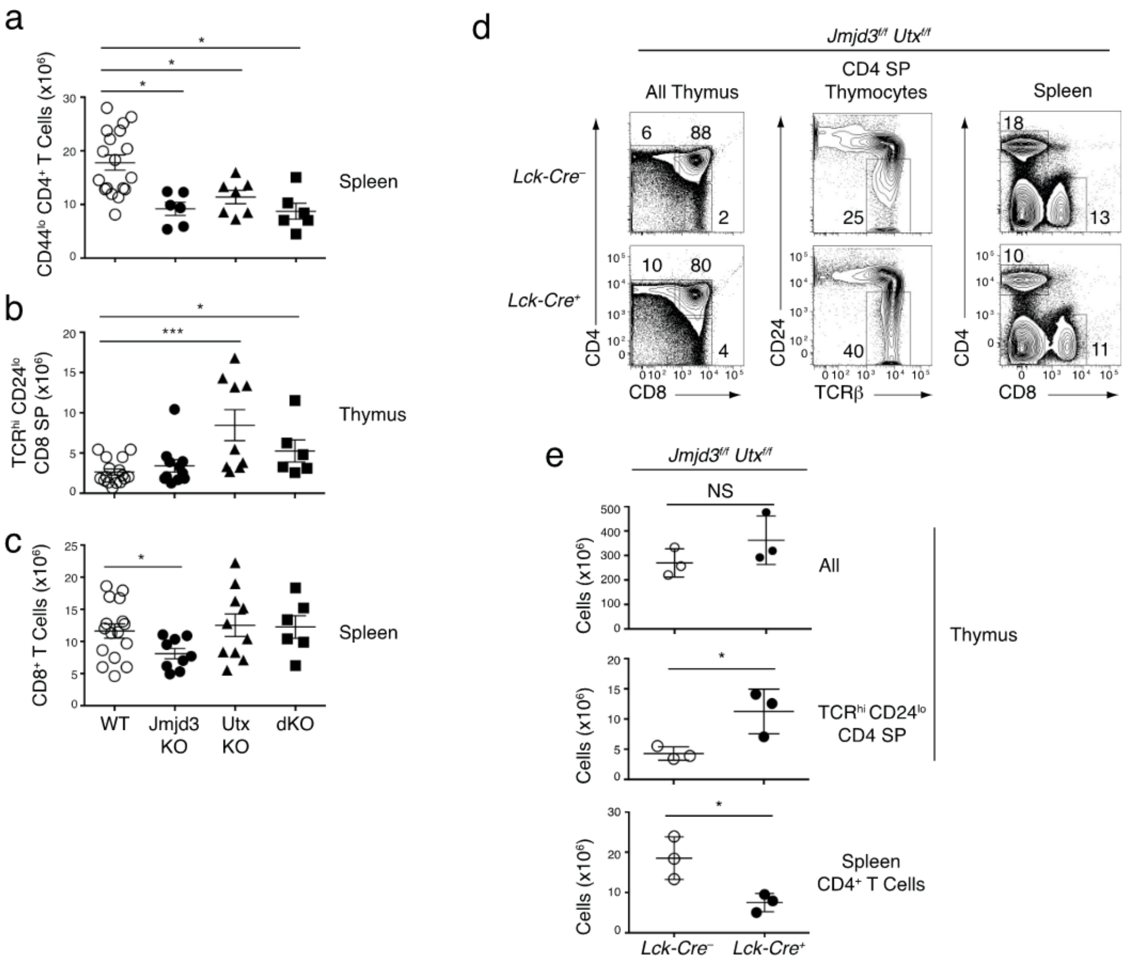

Supplementary Figure 3

Impaired T cell development in H3K27 demethylase-deficient mice.

(**a-c**) Plots indicate numbers of spleen $CD44^{lo}$ $CD4^{+}$ T cells (**a**), mature (TCR^{hi} $CD24^{lo}$) CD8 SP thymocytes (**b**) and spleen $CD8^{+}$ T cells (**c**) from indicated mice. Each symbol represents one 6–8 week-old mouse. Data is from the same mice as in **Fig. 1b**.

(**d**) Contour plots of CD4 and CD8 expression on total thymocytes (left) or splenocytes (right), and TCRβ vs. CD24 expression on CD4 SP thymocytes (middle) from wild-type (WT) or *Jmjd3*$^{f/f}$ *Utx*$^{f/f}$*Lck*-Cre mice.

(**e**) Dot plots show absolute numbers of total or mature thymocytes, or of $CD4^{+}$ T cells from mice in (**d**). Each symbol represents one individual mouse. Data are representative of or combined from three independent experiments (**d, e**).

The statistical significance was determined by unpaired t-test (two-tailed). *: P<0.05, *** P < 0.001, not significant in other cases. Error bars indicate s.d.

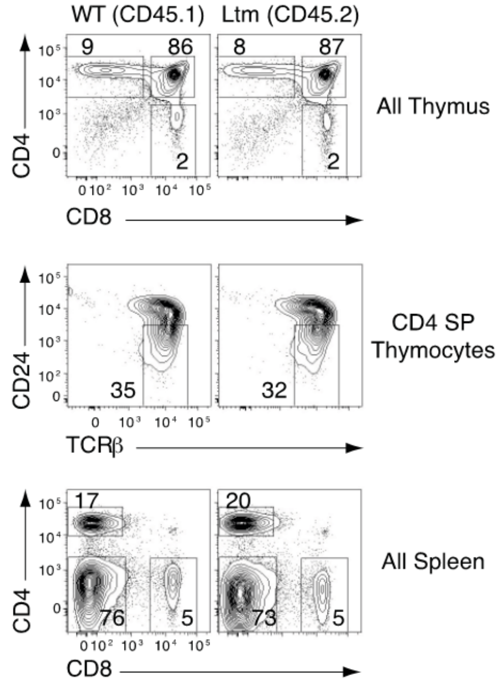

Supplementary Figure 4

T cell development in bone marrow chimeric mice.

Contour plots show expression of CD4 and CD8 in thymocytes (top) or splenocytes (bottom) and TCRβ vs. CD24 (middle) expression in CD4 SP thymocytes from control chimera prepared from littermate control (Ltm, CD45.2) and wild-type (CD45.1) bone marrow. Experiment and mouse numbers indicated in **Figs. 2a, b**.

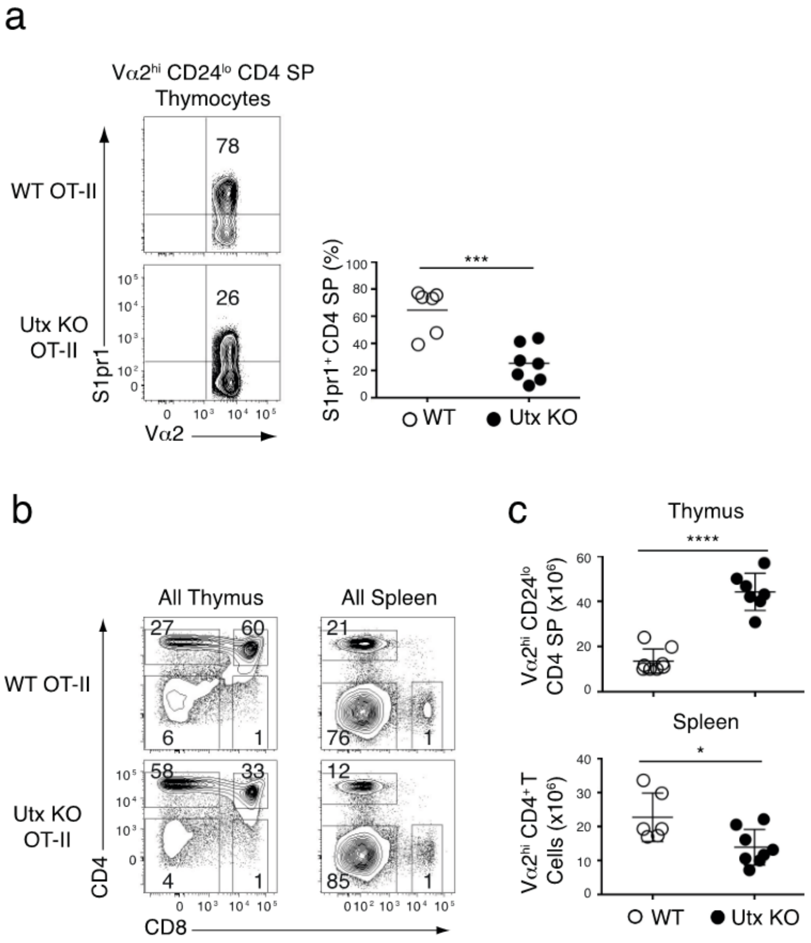

Supplementary Figure 5

Development of Utx-deficient OT-II transgenic T cells.

(**a**) Contour plots show expression of S1pr1 and Vα2 on mature thymocytes and dot plot shows the frequency of $S1pr1^{hi}$ CD4 SP among mature thymocytes from OT-II transgenic wild-type (WT) and Utx KO mice as shown in **Fig. 3b**.

(**b, c**) Contour plots of CD4 and CD8 expression on total thymocytes or splenocytes (**b**) and absolute numbers of mature thymocytes or spleen $V\alpha 2^{hi}$ $CD4^{+}$T cells (**c**) from wild-type (WT) and Utx KO OT-II mice.

Data are representative of or combined from five independent experiments. * $P < 0.05$, *** $P < 0.001$, **** $P < 0.0001$ (unpaired t-test). Error bars indicate s.d.

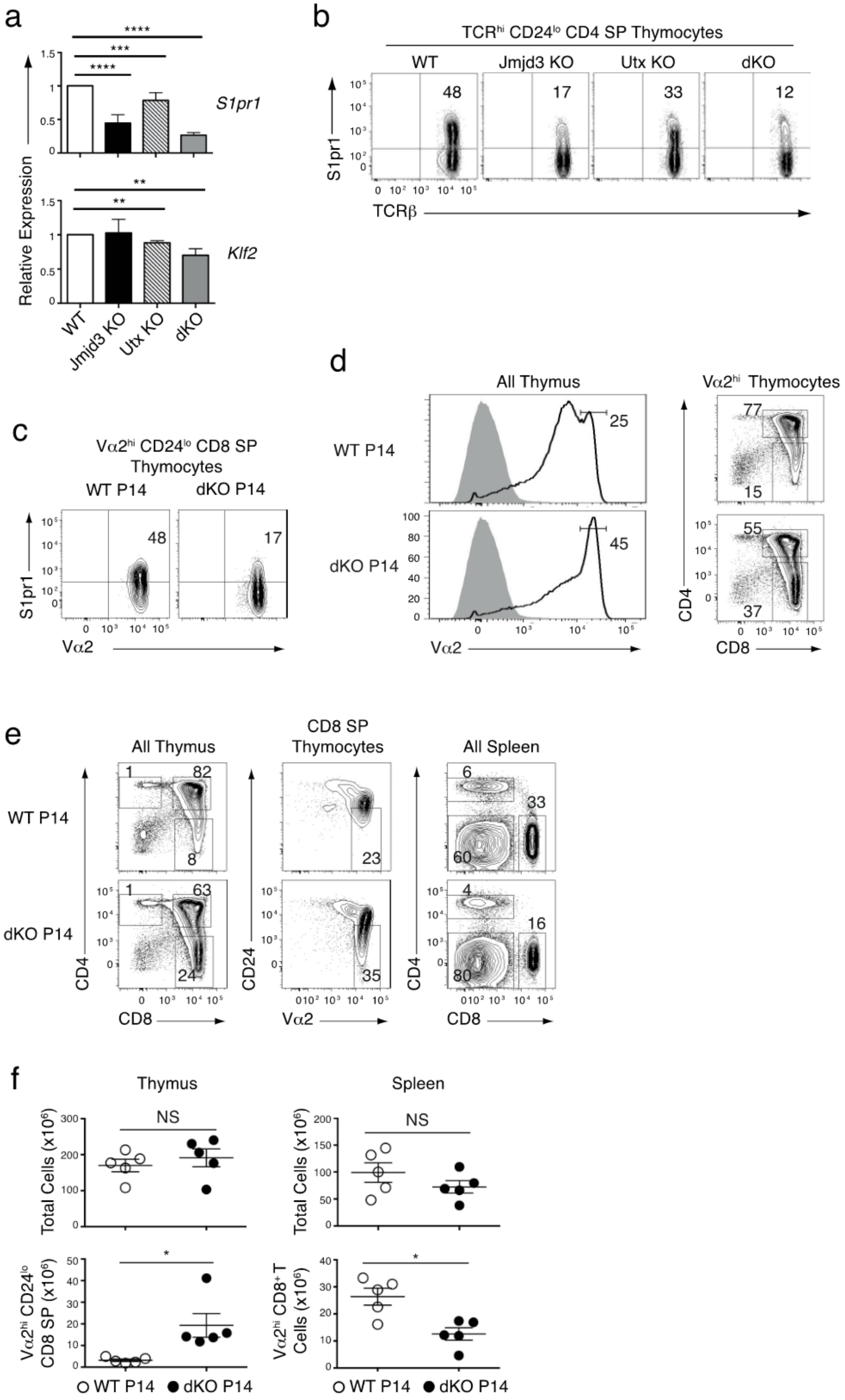
a
Relative Expression
S1pr1
Klf2
WT
Jmjd3 KO
Utx KO
dKO
****
***
****
**
**
b
TCRhi CD24lo CD4 SP Thymocytes
WT
Jmjd3 KO
Utx KO
dKO
48
17
33
12
S1pr1
TCRβ
c
Vα2hi CD24lo CD8 SP Thymocytes
WT P14
dKO P14
48
17
S1pr1
Vα2
d
All Thymus
Vα2hi Thymocytes
WT P14
dKO P14
25
45
77
15
55
37
Vα2
CD4
CD8
e
All Thymus
CD8 SP Thymocytes
All Spleen
WT P14
dKO P14
1
82
8
23
6
33
60
1
63
24
35
4
16
80
CD4
CD8
CD24
Vα2
f
Thymus
Spleen
Total Cells (x10^6)
NS
Vα2hi CD24lo CD8 SP (x10^6)
Vα2hi CD8+ T Cells (x10^6)
*
WT P14
dKO P14

Supplementary Figure 6

Impact of Jmjd3 and Utx on thymocyte development

(**a**) Real-time RT-PCR analysis of S1pr1 and Klf2 expression in $CD24^{lo}$ CD4 SP thymocytes from indicated mice. Bar graphs show fold change relative to wild type values (set as 1) after normalization with 18S rRNA. Data is from three independently sorted sample sets.

(**b**) Contour plots show expression of TCRβ and S1pr1 on TCR^{hi} $CD24^{lo}$ CD4 SP thymocytes of indicated mice.

(**c**) Contour plots show expression of S1pr1 and Vα2 on $V\alpha2^{hi}$ $CD24^{lo}$ CD8 SP thymocytes among mature thymocytes from P14 transgenic wild-type (WT) and dKO mice.

(**d**) Vα2 expression in wild-type (top) and dKO (bottom) thymocytes carrying the P14 TCR transgene (plain line) or in DP thymocytes from non-transgenic wild-type mice (grey shaded). Brackets define the $V\alpha2^{hi}$ subset analyzed for CD4 and CD8 expression (contour plots, right). Numbers on plot indicate percent of cells within nearby box or bracket.

(**e**) Contour plots of CD4 and CD8 expression on total thymocytes (left) or splenocytes (right), and Vα2 vs. CD24 expression on CD8 SP thymocytes (middle) from wild-type (WT) and dKO mice carrying the P14 transgene. Numbers of mice for each genotype indicated in (**f**).

(**f**) Dot plots show absolute numbers of total thymocytes, mature $V\alpha2^{hi}$ $CD24^{lo}$ CD8 SP thymocytes, total splenocytes or $CD8^+$ T cells from mice in (**e**). Each symbol represents one individual mouse.

Data are representative of or combined from three (**b, c,** and **d**) or five (**e, f**) independent determinations on each genotype. The statistical significance was determined by unpaired t-test (two-tailed). *: P<0.05, **: P $<10^{-2}$, ***: P $< 10^{-3}$ and ****: P $< 10^{-4}$. Error bars indicate s.d.

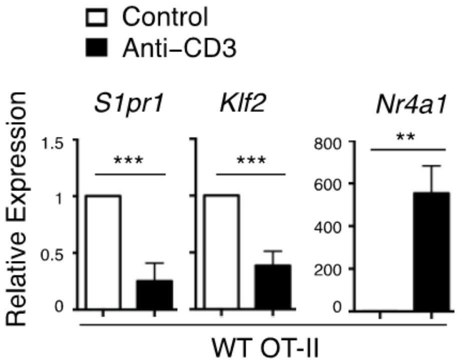

Supplementary Figure 7

TCR engagement represses S1pr1 expression.

Relative expression of *S1pr1, Klf2 and Nr4a1* mRNA in V$\alpha 2^{hi}$ CD24^{lo} CD4 SP thymocytes, from WT OTII mice, after overnight culture with or without anti-CD3. Bar graphs show fold change relative to unstimulated values (set as 1 for each gene) after normalization with 18S rRNA. Data is from three independently sorted sample sets. $^{**}P < 0.01$, and $^{***}P < 0.001$ (unpaired t-test). Error bars indicate s.d.

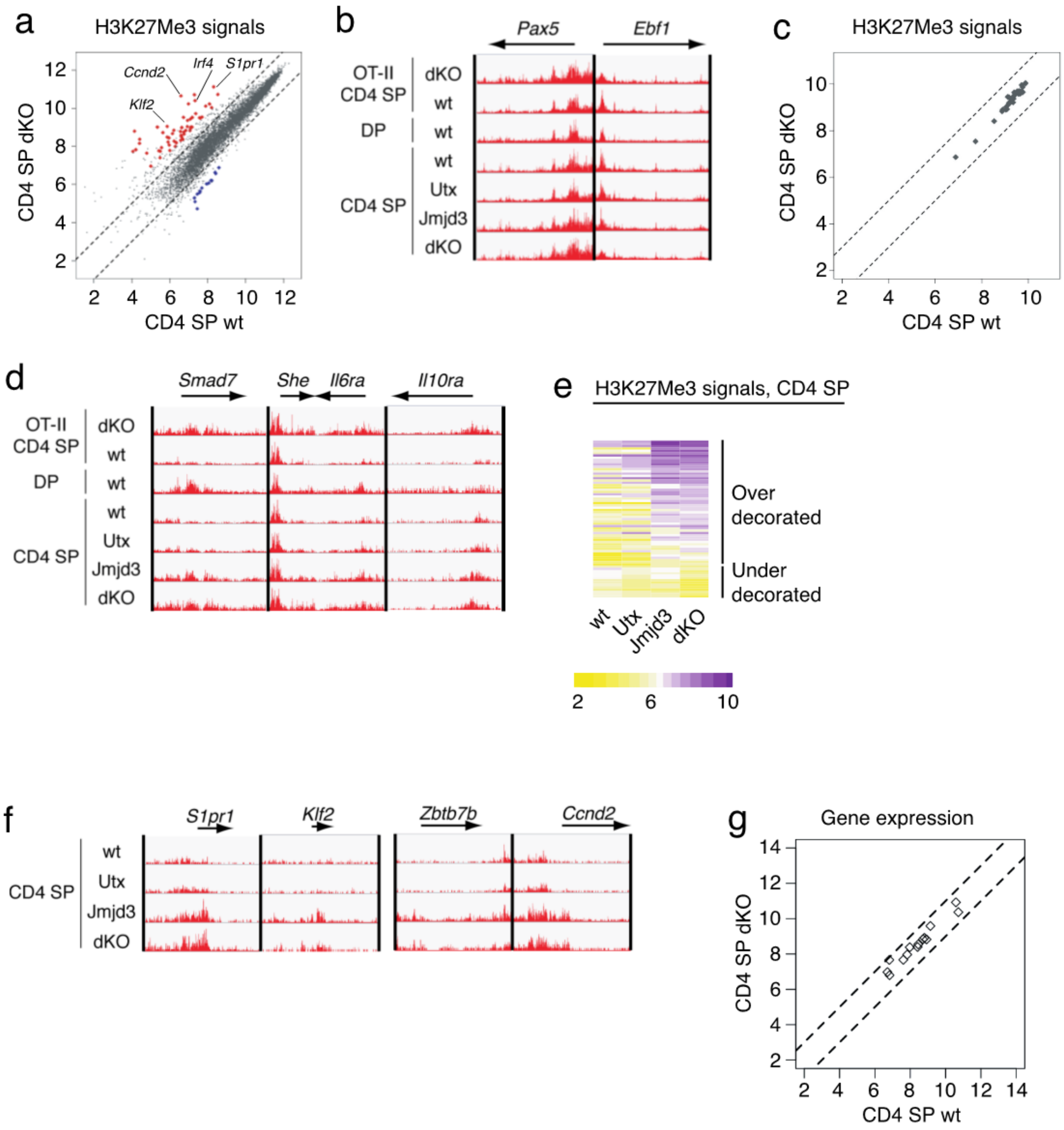

Supplementary Figure 8

Impact of Jmjd3 and Utx on H3K27 trimethylation and gene expression.

(**a**) Scatter plot show H3K27Me3 signals on the 7730 'peak' gene set. Each symbol depicts signal (log_2 value) at a given promoter in wild-type (x-axis) vs. dKO (y-axis) mature CD4 SP OT-II thymocytes. Dotted lines indicate 2-fold changes. Filled red and blue red symbols depict over- and under-decorated genes defined in **Fig. 5b**.

(**b**, **d**) IGV browser tracks show the distribution (normalized sequence reads) of H3K27Me3 ChIPseq signals at indicated loci in wild-type and dKO OT-II transgenic CD4 SP thymocytes (top two tracks), and indicated thymocyte subsets of mice expressing a diverse endogenous TCR repertoire. Arrows indicate transcription boundaries.

(**c**) Scatter plot show H3K27Me3 signals, as in (**a**) on genes within the Hoxa-d clusters in wild-type (x-axis) vs. dKO (y-axis) mature CD4 SP OT-II thymocytes.

(**e**) Heat map indicates ChIPseq signals (log_2 values, color-coding scale at bottom) on over- and under-decorated promoters (defined in **Fig. 5b** and ranked in the same order as in **Fig. 5c**) in mature CD4 SP thymocyte from indicated mice expressing an endogenous diverse TCR repertoire. Each lane represents a separate ChIPseq experiment.

(**f**) IGV browser tracks show the distribution (normalized sequence reads) of H3K27Me3 ChIPseq signals at *S1pr1*, *Klf2*, *Zbtb7b* and *Ccnd2* in thymocytes of mice expressing a diverse endogenous TCR repertoire. Arrows indicate transcription boundaries.

(**g**) Scatter plot show microarray gene expression (log_2 values), on 14 under-decorated genes defined in **Fig. 5b**, in wild-type (x-axis) vs. dKO (y-axis) mature CD4 SP OT-II thymocytes. Dotted lines indicate 2-fold changes.

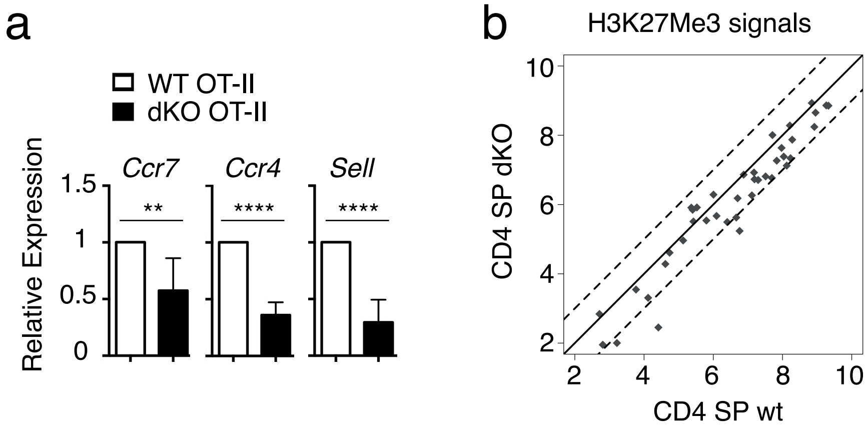

Supplementary Figure 9

Demethylase-independent functions of Jmjd3 and Utx.

(**a**) Relative expression of *Ccr7, Ccr4* and *Sell* in Vα2^{hi} CD24^{lo} CD4 SP thymocytes from indicated mice, as determined by quantitative RT-PCR analyses. Results are presented as fold change relative to wild type values (set as 1) after normalization with 18S. Data is from three independently sorted sample sets. Statistical significance was determined by unpaired t-test. **: $P < 10^{-2}$, ****: $P < 10^{-4}$. Error bars indicate s.d.

(**b**) Scatter plot comparing wild-type vs. dKO H3K27Me3 signals, as in **Supplementary Fig. 8a**, at 41 genes with up-regulated microarray expression in dKO OT-II transgenic CD4 SP thymocytes.

Supplementary Tables

Table S1: 55 over-decorated genes

	H3K27Me3 analysis											Microarray expression								
	Diverse endogenous repertoire					OT-II TCR transgene						OT-II TCR transgene								
Genes	DPwt	CD4SP wt	CD4SP Jmjd3	CD4SP Utx	CD4SP dKO	wt1	wt2	dKO1	dKO2	Signal Ratio (-Log2)	FDR	wt1	wt2	wt3	dKO1	dKO2	dKO3	Expression Ratio (Log2)	Uncorrected p-value	FDR
Fam102a	8.02	3.18	7.18	2.78	6.72	2.11	2.19	6.34	7.25	4.80	0.00	12.07	12.11	12.11	11.90	11.98	11.98	-0.14	0.01	0.07
Ccnd2	9.02	4.31	7.57	3.91	7.20	4.44	4.74	9.20	8.09	4.17	0.00	12.54	12.45	12.32	9.50	9.57	9.71	-2.85	0.00	0.00
Eya2	5.77	4.19	5.66	5.34	6.76	1.96	2.88	6.20	6.54	4.01	0.00	8.50	8.43	8.30	7.24	7.29	7.16	-1.18	0.00	0.01
Zbtb7b	9.05	3.87	6.50	3.90	6.49	2.61	2.22	5.67	6.65	3.84	0.00	9.93	9.99	10.05	9.56	9.80	9.81	-0.27	0.04	0.14
St3gal1	6.46	3.30	6.43	3.55	5.99	1.93	2.21	5.52	5.95	3.77	0.00	6.87	6.85	6.82	6.69	6.65	6.69	-0.17	0.00	0.02
Neurl3	6.90	4.00	5.85	5.74	6.70	3.48	3.23	7.36	6.66	3.71	0.00	7.74	7.86	7.70	6.67	6.49	6.35	-1.26	0.00	0.01
Gfi1	5.30	4.00	4.85	5.28	6.20	2.49	1.94	6.20	5.45	3.66	0.00	7.59	7.56	7.62	7.30	7.26	7.14	-0.36	0.00	0.03
Chl1	8.91	6.76	8.59	7.11	8.50	5.36	5.24	8.64	8.78	3.41	0.00	7.83	8.03	8.08	6.41	6.62	6.59	-1.44	0.00	0.01
Egr2	8.22	5.06	8.04	5.41	7.92	5.24	4.73	8.25	8.21	3.22	0.00	9.74	9.84	9.86	9.50	9.40	9.43	-0.37	0.00	0.02
Irf4	8.11	5.65	8.42	6.08	8.16	5.59	5.08	8.55	8.25	3.05	0.00	9.15	9.16	8.95	7.31	7.12	7.02	-1.94	0.00	0.01
Galnt6	6.71	3.59	5.75	3.33	6.33	4.07	3.22	6.60	6.85	3.01	0.00	10.18	10.23	10.19	10.15	10.10	10.10	-0.08	0.03	0.12
Klf2	7.81	5.19	7.30	5.02	7.03	4.64	3.10	7.22	6.88	2.96	0.01	10.84	11.00	10.94	9.61	9.78	9.80	-1.19	0.00	0.01
Map7d1	5.23	3.16	5.29	3.87	5.60	2.75	2.84	5.53	5.75	2.91	0.01	7.77	7.87	7.95	7.80	7.94	7.94	0.03	0.72	0.85
S1pr1	8.72	6.88	8.86	7.43	8.89	6.40	6.23	9.38	8.86	2.83	0.00	11.41	11.54	11.67	7.37	7.18	7.21	-4.29	0.00	0.00
Fgr	6.07	4.67	6.73	6.31	7.34	5.12	4.33	7.86	7.17	2.77	0.01	6.17	6.24	6.13	5.93	6.04	6.01	-0.19	0.01	0.07
H2-Q2	5.43	5.88	6.44	6.25	6.84	4.51	3.93	7.43	6.26	2.70	0.02	7.55	7.60	7.38	7.25	7.20	7.13	-0.31	0.01	0.08
Ndrg1	6.23	5.13	6.29	4.62	6.28	3.98	3.76	6.65	6.31	2.63	0.00	10.13	10.22	10.29	9.79	9.75	9.74	-0.45	0.00	0.02
E2f2	5.35	4.85	6.43	5.06	6.02	4.58	3.72	6.97	6.38	2.48	0.01	7.54	7.46	7.34	6.82	6.81	6.82	-0.63	0.00	0.01
Gadd45g	7.13	4.56	7.06	5.12	6.61	4.85	4.42	7.63	6.34	2.47	0.03	5.52	5.75	5.87	5.68	5.71	5.52	-0.08	0.54	0.72
D8Ertd82e	7.44	7.16	8.19	7.56	7.83	5.25	4.62	7.58	7.14	2.40	0.01	9.32	9.28	9.34	9.74	10.01	10.12	0.64	0.01	0.04
Nr4a1	6.01	4.10	6.06	4.50	6.33	4.44	3.49	6.55	6.34	2.39	0.01	9.88	10.19	10.44	9.72	9.51	9.30	-0.66	0.03	0.12
Foxp4	6.12	3.23	5.46	3.70	5.12	3.62	3.23	6.14	5.40	2.38	0.02	8.91	8.93	8.91	8.90	8.90	8.91	-0.02	0.09	0.25
Egln3	6.07	6.49	7.31	6.89	7.38	5.15	5.23	7.56	7.47	2.34	0.00	7.48	7.73	7.74	5.98	6.96	7.40	-0.87	0.11	0.28
Tnfrsf1b	6.35	5.03	6.92	5.88	6.86	5.36	4.81	7.58	7.29	2.33	0.01	8.58	8.61	8.58	7.84	7.64	7.65	-0.88	0.00	0.01
Il10ra	6.55	6.46	7.70	6.97	7.88	5.70	5.03	7.91	7.53	2.32	0.01	8.13	8.68	8.89	5.78	5.69	5.82	-2.80	0.00	0.01
Nbeal2	5.95	4.82	5.38	5.58	6.08	4.10	3.05	6.33	5.53	2.30	0.03	9.18	9.21	9.17	8.04	8.19	8.26	-1.02	0.00	0.01
Prkch	5.92	2.73	5.85	3.99	5.58	3.60	3.42	5.76	5.73	2.25	0.01	11.37	11.36	11.40	11.18	11.22	11.20	-0.18	0.00	0.01
Alox5ap	5.81	5.04	5.90	5.20	6.26	4.50	3.33	6.48	5.98	2.20	0.03	6.19	6.14	6.13	5.70	5.87	5.50	-0.46	0.01	0.08
Trib1	5.40	5.09	6.42	5.03	6.36	5.02	4.11	7.20	6.36	2.20	0.03	6.49	6.61	6.67	6.56	6.44	6.69	-0.03	0.76	0.87
Lfng	9.16	7.00	8.94	6.83	8.64	6.65	6.42	8.58	8.87	2.19	0.01	11.11	11.21	11.21	10.67	10.82	10.84	-0.40	0.00	0.04
Dpp4	6.07	6.26	7.47	6.66	7.50	4.98	4.88	7.43	6.65	2.17	0.02	10.32	10.19	10.21	8.70	8.49	8.86	-1.56	0.00	0.01
Edaradd	5.79	7.62	8.24	7.93	8.12	6.32	5.87	8.23	8.22	2.11	0.01	6.98	7.36	7.36	6.07	6.07	6.16	-1.14	0.00	0.02
Ubash3b	8.23	5.93	7.09	6.71	7.66	6.47	5.31	8.15	8.07	2.10	0.03	9.02	9.13	9.08	6.64	6.89	7.00	-2.23	0.00	0.00
Art4	5.10	6.52	5.46	7.38	7.36	4.05	4.42	6.21	6.35	2.06	0.01	8.17	8.12	8.21	7.51	7.48	7.57	-0.64	0.00	0.01
Extl3	5.99	5.32	6.04	5.40	6.14	4.41	3.54	6.36	5.78	2.04	0.03	9.81	9.90	10.02	9.70	9.71	9.73	-0.20	0.03	0.13
Tbc1d1	7.78	5.12	6.31	5.32	6.51	4.91	4.78	7.17	6.51	2.03	0.02	9.22	9.19	9.18	8.36	8.31	8.39	-0.85	0.00	0.00
Cass4	5.84	5.92	6.48	5.63	6.51	4.78	4.70	6.59	6.91	2.02	0.01	5.79	5.75	5.75	5.63	5.72	5.80	-0.04	0.46	0.66
Snai3	6.37	5.30	6.34	5.71	6.23	4.94	4.26	6.92	6.25	1.98	0.03	5.73	5.88	5.75	5.32	5.22	5.37	-0.48	0.00	0.02
Card6	5.81	3.38	5.31	3.50	5.30	3.13	2.89	4.77	5.15	1.97	0.05	10.06	10.06	10.02	10.38	10.36	10.43	0.35	0.00	0.01
D930028M14Rik	6.47	5.71	6.98	6.09	6.53	5.80	5.33	7.87	7.13	1.96	0.03	6.31	6.35	6.41	6.24	6.27	6.26	-0.10	0.03	0.13
Cyfip2	5.37	4.09	5.29	4.64	4.92	3.45	3.54	5.17	5.63	1.96	0.04	11.40	11.44	11.44	11.29	11.34	11.39	-0.09	0.05	0.17
Nrp1	6.02	6.83	8.08	6.78	7.42	5.78	5.62	8.11	6.96	1.95	0.04	7.14	7.05	7.00	6.30	6.44	6.49	-0.65	0.00	0.02
Kbtbd11	7.89	5.79	6.95	4.86	6.22	4.99	4.53	6.81	6.47	1.87	0.03	9.51	9.52	9.45	9.17	9.29	9.30	-0.24	0.01	0.05
Hectd2	6.36	5.44	6.21	6.20	6.59	4.90	4.35	6.47	6.55	1.86	0.03	7.86	7.92	7.65	6.18	6.37	6.33	-1.52	0.00	0.01
4930546C10Rik	5.25	4.18	5.88	4.98	6.51	5.48	4.75	7.25	6.77	1.86	0.03	6.07	6.01	5.79	5.69	5.71	5.85	-0.20	0.11	0.28
Unc93b1	6.73	6.74	7.92	6.61	7.41	5.87	5.36	7.63	7.35	1.85	0.03	6.11	6.05	6.12	5.90	5.94	5.78	-0.22	0.01	0.08
Pim1	5.32	4.67	6.63	5.34	6.47	4.58	4.12	6.32	6.06	1.83	0.03	8.89	8.82	8.68	7.46	7.66	7.57	-1.24	0.00	0.01
Cnn3	6.60	6.74	7.22	6.44	6.78	5.31	4.88	7.02	6.85	1.83	0.02	9.39	9.40	9.31	9.19	9.26	9.49	-0.05	0.62	0.77
Ccr2	6.19	6.28	7.74	6.76	7.86	6.54	5.88	8.31	7.75	1.81	0.04	4.22	4.20	4.20	4.16	4.12	4.15	-0.06	0.01	0.06
Tgif1	4.89	5.42	5.62	6.36	6.37	3.77	4.25	5.99	5.58	1.80	0.04	8.12	8.06	7.93	8.10	8.08	8.05	0.04	0.53	0.71
8030423F21Rik	6.07	5.05	6.61	5.91	6.83	5.21	4.93	7.12	6.53	1.78	0.03	4.69	4.73	4.72	4.77	4.80	4.79	0.07	0.01	0.07
Trpm1	6.60	5.49	6.95	5.45	6.79	5.53	4.83	7.04	6.98	1.78	0.03	5.19	5.20	5.22	5.04	5.11	5.17	-0.09	0.07	0.20
Nipal1	5.34	4.51	5.72	4.96	5.80	4.71	4.46	6.29	6.35	1.73	0.03	7.93	8.08	8.17	7.46	7.67	7.78	-0.42	0.02	0.10
Foxo1	6.25	4.28	6.11	4.99	5.49	4.36	4.23	6.21	5.78	1.72	0.04	11.07	11.18	11.10	10.11	10.19	10.24	-0.94	0.00	0.01
Ncald	6.41	6.87	6.91	6.86	7.22	4.76	4.63	6.43	6.33	1.69	0.03	6.87	7.10	6.95	6.61	6.73	6.83	-0.25	0.05	0.17

Supplementary Table 1: Over-decorated genes.

The table lists the 55 genes 'over-decorated' with H3K27Me3 in mature dKO OT-II CD4 SP thymocytes, as defined in Fig. 5b. Genes are ranked by decreasing H3K27Me3 signal ratio.

Table S2: Under-expressed genes

	H3K27Me3 analysis													Microarray expression							
	Diverse endogenous repertoire					OT-II TCR transgene							OT-II TCR transgene							
Genes	DP wt	CD4SP wt	CD4SP Jmjd3	CD4SP Utx	CD4SP dKO	wt1	wt2	dKO1	dKO2	Signal Ratio (-Log2)	uncorrected p-value	FDR	wt1	wt2	wt3	dKO1	dKO2	dKO3	Expression Ratio (Log2)	FDR
Ccnd2	9.02	4.31	7.57	3.91	7.20	4.44	4.74	9.20	8.09	4.17	0.00	0.00	12.54	12.45	12.32	9.50	9.57	9.71	-2.85	0.00
Eya2	5.77	4.19	5.66	5.34	6.76	1.96	2.88	6.20	6.54	4.01	0.00	0.00	8.50	8.43	8.30	7.24	7.29	7.16	-1.18	0.01
Neurl3	6.90	4.00	5.85	5.74	6.70	3.48	3.23	7.36	6.66	3.71	0.00	0.00	7.74	7.86	7.70	6.67	6.49	6.35	-1.26	0.01
Itgb7	6.14	3.39	5.29	4.01	5.12	2.44	1.73	5.97	5.34	3.60	0.00	0.00	10.29	10.32	10.30	8.82	8.80	8.65	-1.55	0.00
Chl1	8.91	6.76	8.59	7.11	8.50	5.36	5.24	8.64	8.78	3.41	0.00	0.00	7.83	8.03	8.08	6.41	6.62	6.59	-1.44	0.01
Ccr8	5.45	3.89	4.53	3.75	4.71	3.19	2.52	6.40	5.35	3.10	0.00	0.01	8.34	8.47	8.82	7.40	6.92	6.60	-1.57	0.04
Irf4	8.11	5.65	8.42	6.08	8.16	5.59	5.08	8.55	8.25	3.05	0.00	0.00	9.15	9.16	8.95	7.31	7.12	7.02	-1.94	0.01
Klf2	7.81	5.19	7.30	5.02	7.03	4.64	3.10	7.22	6.88	2.96	0.00	0.01	10.84	11.00	10.94	9.61	9.78	9.80	-1.19	0.01
S1pr1	8.72	6.88	8.86	7.43	8.89	6.40	6.23	9.38	8.86	2.83	0.00	0.00	11.41	11.54	11.67	7.37	7.18	7.21	-4.29	0.00
Bcl2	6.67	4.03	5.41	4.18	5.00	3.63	2.97	6.12	5.68	2.57	0.00	0.01	11.74	11.75	11.77	10.47	10.64	10.85	-1.10	0.01
Il10ra	6.55	6.46	7.70	6.97	7.88	5.70	5.03	7.91	7.53	2.32	0.00	0.01	8.13	8.68	8.89	5.78	5.69	5.82	-2.80	0.01
Nbeal2	5.95	4.82	5.38	5.58	6.08	4.10	3.05	6.33	5.53	2.30	0.00	0.03	9.18	9.21	9.17	8.04	8.19	8.26	-1.02	0.01
Ptgir	5.19	4.03	4.59	2.87	3.55	2.72	1.70	5.05	3.68	2.28	0.00	0.19	8.01	8.16	8.15	6.98	6.89	6.67	-1.26	0.01
D830046C22	6.73	5.44	4.31	4.35	4.52	3.07	2.98	5.67	4.70	2.26	0.00	0.04	6.60	6.64	6.61	5.24	5.36	5.09	-1.39	0.01
Dpp4	6.07	6.26	7.47	6.66	7.50	4.98	4.88	7.43	6.65	2.17	0.00	0.02	10.32	10.19	10.21	8.70	8.49	8.86	-1.56	0.01
Edaradd	5.79	7.62	8.24	7.93	8.12	6.32	5.87	8.23	8.22	2.11	0.00	0.01	6.98	7.36	7.36	6.07	6.07	6.16	-1.14	0.02
Ubash3b	8.23	5.93	7.09	6.71	7.66	6.47	5.31	8.15	8.07	2.10	0.00	0.03	9.02	9.13	9.08	6.64	6.89	7.00	-2.23	0.00
P2rx7	6.11	5.35	6.10	5.62	6.27	4.87	3.82	6.98	5.88	2.08	0.00	0.07	6.80	7.10	7.20	5.86	5.83	5.73	-1.22	0.01
Itgb3	5.76	4.18	4.95	5.39	5.38	2.23	3.27	5.14	4.15	2.01	0.00	0.15	10.92	10.98	10.95	8.81	9.22	9.25	-1.85	0.01
Hectd2	6.36	5.44	6.21	6.20	6.59	4.90	4.35	6.47	6.55	1.86	0.00	0.03	7.86	7.92	7.65	6.18	6.37	6.33	-1.52	0.01
Pim1	5.32	4.67	6.63	5.34	6.47	4.58	4.12	6.32	6.06	1.83	0.00	0.03	8.89	8.82	8.68	7.46	7.66	7.57	-1.24	0.01
Sh3bp5	7.21	6.41	7.47	7.22	7.29	6.26	5.87	8.00	7.36	1.64	0.00	0.07	9.13	9.14	9.09	7.80	7.63	7.88	-1.35	0.01
Aqp9	5.39	3.65	4.86	4.44	4.87	3.50	3.01	5.04	4.72	1.63	0.00	0.12	6.48	6.42	6.38	4.99	5.16	5.09	-1.35	0.00
Sytl2	6.99	6.48	6.91	6.50	7.30	6.29	5.49	7.58	7.41	1.55	0.00	0.10	7.90	8.11	8.32	5.98	5.92	6.14	-2.10	0.01
Cd44	7.89	6.36	8.18	7.44	8.31	6.38	6.02	7.85	7.65	1.54	0.00	0.11	7.79	7.90	8.00	6.80	6.87	6.76	-1.09	0.01
Ppp3cc	6.52	5.49	7.03	5.84	7.06	5.98	5.59	7.18	7.32	1.45	0.00	0.14	8.68	8.79	8.56	7.12	6.94	6.96	-1.66	0.01
Adamts3	6.83	6.42	7.06	6.39	7.04	6.24	5.79	7.63	7.17	1.38	0.00	0.20	7.64	7.66	7.65	6.06	6.00	6.15	-1.58	0.00
Samhd1	9.21	5.12	7.90	4.94	7.35	7.36	6.88	8.57	8.42	1.36	0.01	0.36	11.52	11.37	11.13	9.39	9.14	9.16	-2.11	0.01
Il18r1	6.93	7.07	8.17	7.59	8.23	6.82	6.68	8.24	7.95	1.35	0.01	0.34	7.50	7.64	7.46	5.30	5.17	5.34	-2.26	0.00
Smad7	8.31	6.38	7.49	6.77	7.65	6.53	6.19	7.69	7.67	1.31	0.01	0.28	10.16	10.08	10.04	8.95	9.08	9.12	-1.05	0.01
St3gal6	8.25	7.36	7.88	8.09	7.96	6.59	7.03	8.05	8.18	1.30	0.01	0.38	9.39	9.45	9.45	8.24	8.23	8.27	-1.19	0.00
Crip1	6.14	5.88	6.82	6.42	6.84	6.48	5.34	7.81	6.60	1.30	0.02	0.47	11.32	11.23	11.16	9.67	9.63	9.51	-1.63	0.00
Tdrp	8.18	6.22	7.78	6.74	7.55	7.20	7.34	8.57	8.39	1.21	0.02	0.55	8.77	8.81	8.73	7.92	7.73	7.49	-1.06	0.02
Igfbp4	9.27	7.93	8.78	7.18	8.17	8.29	7.82	9.33	9.09	1.14	0.04	0.73	10.53	10.28	9.70	6.28	6.46	6.42	-3.78	0.01
Btbd11	7.85	7.80	8.17	8.37	8.14	7.34	6.90	8.45	8.07	1.14	0.03	0.61	9.14	9.12	9.09	6.41	6.34	6.30	-2.76	0.00
Xkrx	5.94	5.79	5.44	6.05	6.57	5.49	5.19	6.32	6.46	1.04	0.02	0.55	8.65	8.71	8.90	6.95	7.26	7.29	-1.59	0.01
B4galt5	7.34	5.77	7.60	6.54	7.25	6.58	6.16	7.41	7.43	1.03	0.03	0.67	9.28	9.27	9.12	8.13	7.95	8.15	-1.15	0.01
Cd9	7.80	7.36	8.11	8.03	8.13	7.55	6.97	8.43	7.87	0.89	0.09	1.00	8.84	8.66	8.39	7.44	7.36	7.34	-1.25	0.02
Sidt1	6.11	6.21	6.83	6.10	6.39	6.42	6.33	7.36	7.10	0.86	0.07	0.94	8.99	8.91	8.70	6.86	6.88	6.78	-2.03	0.00
Gpr97	6.16	5.70	5.71	5.54	5.62	6.17	5.27	6.91	6.16	0.79	0.09	1.00	8.87	8.98	8.95	7.93	7.93	7.86	-1.03	0.00
Ckb	9.40	8.68	9.19	8.76	9.13	8.93	8.49	9.75	9.06	0.72	0.18	1.00	9.86	10.00	10.13	7.77	7.86	7.89	-2.16	0.00
Rnf144a	6.05	7.05	7.64	7.25	7.50	7.51	6.97	8.13	7.81	0.72	0.17	1.00	8.58	8.54	8.49	7.44	7.44	7.33	-1.14	0.00
Sgk1	8.74	8.76	9.15	8.76	9.20	8.59	8.52	9.11	9.25	0.63	0.25	1.00	8.17	8.08	8.06	6.99	6.98	7.01	-1.11	0.00
Klhdc2	6.13	6.29	7.23	6.42	6.63	7.31	6.23	7.74	7.18	0.61	0.22	1.00	7.63	7.47	7.38	5.79	5.81	6.01	-1.62	0.01
Cish	8.12	6.06	7.06	5.43	6.38	7.27	6.83	7.87	7.45	0.60	0.23	1.00	7.84	7.58	7.47	6.50	6.66	6.63	-1.03	0.02
Map3k5	7.19	6.89	7.53	7.45	7.76	7.18	7.06	7.81	7.61	0.59	0.24	1.00	8.15	8.23	8.10	6.51	6.61	6.46	-1.63	0.00
Tnfrsf25	8.75	7.36	8.16	7.99	8.14	8.58	8.07	9.25	8.50	0.57	0.30	1.00	8.23	8.30	8.17	6.84	6.72	6.73	-1.47	0.00
St8sia6	9.19	7.96	8.89	7.96	8.47	8.48	8.11	8.79	8.95	0.56	0.30	1.00	10.24	10.07	10.13	8.78	8.96	9.10	-1.20	0.01
Cacnb3	8.89	8.98	9.29	9.23	9.40	9.60	9.28	10.30	9.64	0.56	0.27	1.00	7.76	7.40	7.43	6.51	6.54	6.50	-1.01	0.02
Ar	8.44	7.94	8.37	8.35	8.53	8.39	7.92	8.92	8.50	0.55	0.31	1.00	8.22	8.33	8.18	6.65	6.63	6.80	-1.55	0.00
Aff3	5.58	6.50	6.63	6.39	6.70	6.50	6.26	7.22	6.54	0.53	0.26	1.00	10.44	10.43	10.45	9.31	9.44	9.56	-1.01	0.01
Sp6	8.48	8.10	8.47	8.28	8.66	8.42	7.95	8.87	8.59	0.53	0.33	1.00	7.15	7.30	7.25	5.98	6.16	5.78	-1.26	0.01
Prr13	6.07	4.24	4.64	4.86	4.93	4.25	3.92	4.80	4.45	0.53	0.21	1.00	7.87	7.96	7.82	6.65	6.87	6.98	-1.05	0.01
Myo10	6.00	6.99	7.54	6.86	7.36	7.21	7.07	7.75	7.59	0.53	0.29	1.00	9.08	9.06	9.03	7.56	7.52	7.60	-1.50	0.00
Cers6	6.29	7.55	7.86	7.05	7.42	7.28	6.92	7.77	7.27	0.43	0.40	1.00	9.56	9.65	9.71	8.24	8.46	8.61	-1.21	0.01
Art2b	4.92	5.62	5.29	4.67	5.40	5.35	5.09	5.61	5.53	0.34	0.41	1.00	7.22	7.38	7.29	5.28	5.00	4.49	-2.37	0.01
Man1c1	8.35	8.79	9.00	8.67	8.76	8.90	8.32	9.20	8.72	0.34	0.54	1.00	8.27	8.06	7.70	6.71	6.71	6.90	-1.23	0.03

Table S2: Under-expressed genes

Gramd3	8.62	8.61	9.17	8.76	8.67	8.53	8.58	8.78	8.98	0.32	0.55	1.00	9.71	9.61	9.49	8.36	7.86	7.69	-1.64	0.02
Cd55	6.33	6.39	6.67	6.59	6.70	6.46	6.11	6.65	6.59	0.32	0.49	1.00	6.69	6.48	6.19	5.07	5.46	5.30	-1.18	0.03
Lypd6b	9.04	9.06	9.53	9.18	9.28	9.28	9.07	9.51	9.44	0.29	0.57	1.00	8.35	8.37	8.31	6.46	6.33	6.33	-1.97	0.00
Aim1	9.09	8.56	9.19	8.54	8.75	8.97	8.73	9.27	8.92	0.25	0.64	1.00	10.11	10.09	10.08	8.99	9.03	9.25	-1.01	0.01
Nav2	9.42	9.55	9.53	9.34	9.73	9.45	9.48	9.50	9.75	0.16	0.74	1.00	8.23	8.07	7.74	6.35	6.49	6.47	-1.57	0.01
Tnfaip8l1	5.41	5.21	5.40	5.09	5.49	5.65	5.12	5.95	5.01	0.14	0.73	1.00	7.95	7.84	7.74	6.54	6.75	6.56	-1.22	0.01
Tlr7	6.11	5.82	5.72	6.42	6.64	5.84	6.20	5.69	6.53	0.14	0.76	1.00	8.51	8.51	8.75	7.69	7.49	7.46	-1.05	0.02
Tmem64	4.82	4.39	4.55	4.39	4.51	3.77	3.92	3.84	4.10	0.13	0.78	1.00	10.57	10.63	10.69	9.48	9.64	9.59	-1.06	0.01
Macf1	9.15	9.69	9.58	9.51	9.49	9.38	9.38	9.36	9.56	0.08	0.87	1.00	10.54	10.51	10.49	9.46	9.45	9.52	-1.03	0.00
Dpy19l1	5.06	5.21	5.59	5.46	5.19	5.42	5.32	5.29	5.52	0.03	0.94	1.00	7.64	7.53	7.45	6.47	6.16	6.36	-1.21	0.01
Nfix	8.85	9.18	9.07	8.63	8.44	8.63	8.37	8.79	8.05	-0.05	0.93	1.00	8.21	8.25	8.02	6.71	6.70	6.89	-1.39	0.01
Fry	6.94	7.48	7.72	7.03	7.18	6.89	7.06	6.25	7.02	-0.29	0.55	1.00	9.14	9.03	9.07	6.92	7.04	7.19	-2.03	0.00
4931406H21	3.92	3.78	4.24	3.72	2.82	3.05	4.60	4.56	3.93	No Peak	N/A	N/A	7.22	7.19	7.02	5.47	5.73	5.72	-1.50	0.01
Emp3	4.49	3.49	3.84	4.22	4.02	2.95	3.11	4.17	3.21	No Peak	N/A	N/A	11.91	11.91	11.90	10.29	10.59	10.63	-1.40	0.01
Airn	5.30	4.74	4.68	5.14	5.37	4.69	3.91	5.12	4.64	No Peak	N/A	N/A	9.13	9.29	9.41	7.88	7.90	8.07	-1.33	0.01
Pole2	3.71	3.62	4.31	3.79	3.79	3.62	3.56	4.46	4.06	No Peak	N/A	N/A	8.16	8.04	7.86	6.62	6.70	6.81	-1.31	0.01
Tnfsf8	4.40	4.09	4.14	4.35	4.56	3.14	2.81	4.50	5.04	No Peak	N/A	N/A	7.37	6.98	7.04	5.12	5.35	5.38	-1.85	0.01
Fam111a	4.08	3.18	2.98	3.51	4.32	2.50	1.90	3.81	4.52	No Peak	N/A	N/A	7.26	7.20	7.18	5.87	5.75	5.41	-1.54	0.01
Ecm1	3.77	3.76	3.01	3.29	3.42	3.40	3.34	3.68	3.46	No Peak	N/A	N/A	7.76	7.93	8.11	6.72	6.78	6.82	-1.16	0.01
Adk	5.45	5.97	5.96	5.76	6.10	5.74	5.29	5.86	5.74	No Peak	N/A	N/A	8.24	8.28	8.43	7.23	7.40	7.13	-1.07	0.01
Phyhd1	4.29	4.30	4.51	3.62	3.25	3.34	2.89	3.83	4.03	No Peak	N/A	N/A	7.46	7.36	7.21	6.28	6.11	6.34	-1.09	0.01
Anxa2	5.20	6.10	6.91	5.95	6.15	5.58	5.06	6.66	6.00	No Peak	N/A	N/A	7.79	8.01	7.87	6.91	6.81	6.65	-1.10	0.01
Itih5	5.04	3.53	4.48	4.91	4.80	4.15	3.56	4.81	5.21	No Peak	N/A	N/A	6.69	6.83	7.27	5.03	5.08	5.20	-1.82	0.01
Stat4	5.08	3.73	5.22	5.09	5.55	2.96	0.66	5.14	5.09	No Peak	N/A	N/A	9.90	10.08	10.29	8.91	8.83	9.00	-1.18	0.02
Dennd2c	4.25	4.95	4.91	4.85	5.02	4.63	4.77	5.02	4.30	No Peak	N/A	N/A	6.87	6.92	6.79	5.66	5.87	5.99	-1.02	0.02
Ly6c2	3.17	3.75	4.49	3.71	4.99	4.13	3.61	4.91	4.72	No Peak	N/A	N/A	8.47	8.31	7.79	6.25	6.34	6.16	-1.94	0.02
Hsd11b1	3.25	2.90	3.88	3.36	2.96	2.34	1.42	2.51	3.23	No Peak	N/A	N/A	9.71	9.70	9.71	8.82	8.49	8.42	-1.13	0.02
Tnfrsf4	4.18	3.40	3.74	2.72	1.85	0.77	2.20	3.76	3.03	No Peak	N/A	N/A	8.47	8.41	8.49	7.59	7.25	7.17	-1.12	0.02
Phxr4	3.42	3.31	4.60	3.06	3.68	3.56	3.26	4.32	4.60	No Peak	N/A	N/A	9.65	9.62	9.60	7.54	8.05	8.26	-1.67	0.02
Ms4a4c	3.24	2.79	3.14	3.41	4.61	2.80	3.31	4.97	4.95	No Peak	N/A	N/A	7.19	6.92	6.90	6.17	5.87	5.83	-1.05	0.03
AW112010	2.85	3.23	2.70	3.39	4.39	2.88	3.05	3.43	4.09	No Peak	N/A	N/A	8.91	8.76	8.45	7.70	7.42	7.33	-1.23	0.03
Wnk1	2.57	1.61	3.05	3.10	2.44	1.24	-0.46	2.45	-0.75	No Peak	N/A	N/A	9.16	9.18	9.06	7.78	8.08	8.32	-1.07	0.03
Tbrg3	2.46	2.17	2.25	1.73	2.18	0.93	2.37	0.44	1.71	No Peak	N/A	N/A	8.85	8.81	8.71	7.38	7.71	7.95	-1.11	0.03
Gvin1	0.69	-0.76	-0.86	1.22	-1.06	0.28	0.56	-0.26	1.13	No Peak	N/A	N/A	8.46	8.39	8.35	6.87	6.91	6.01	-1.81	0.04
Ampd1	4.39	4.59	5.06	4.47	5.27	4.11	4.50	4.16	4.83	No Peak	N/A	N/A	6.74	7.06	6.38	5.53	5.23	5.52	-1.30	0.04
Sp100	3.24	2.01	2.01	2.85	2.95	4.21	2.99	3.96	3.91	No Peak	N/A	N/A	8.60	8.59	8.63	7.26	7.59	7.87	-1.03	0.04
Oas3	4.32	3.66	4.10	3.31	3.25	2.40	2.05	3.61	3.48	No Peak	N/A	N/A	8.63	8.65	8.44	7.81	7.45	7.20	-1.09	0.04
Dapl1	4.63	4.23	4.25	4.17	5.34	3.25	3.59	5.47	4.85	No Peak	N/A	N/A	9.27	9.32	9.12	5.14	5.10	4.95	-4.17	0.00
Cd97	4.16	1.34	2.77	3.06	3.45	2.42	2.06	3.54	4.05	No Peak	N/A	N/A	11.03	11.04	11.07	10.02	10.02	10.07	-1.01	0.00
Flt3l	4.93	3.04	4.34	3.97	3.94	1.76	3.02	4.13	3.86	No Peak	N/A	N/A	10.13	10.14	10.17	8.90	8.89	8.92	-1.24	0.00
Trat1	2.32	1.65	3.67	3.52	1.94	2.21	1.68	2.07	2.53	No Peak	N/A	N/A	9.57	9.61	9.69	7.86	7.95	8.05	-1.67	0.00
Lgals1	4.91	3.17	3.99	3.65	4.57	2.96	2.98	5.38	4.10	No Peak	N/A	N/A	10.76	10.74	10.63	8.07	7.90	7.70	-2.82	0.00
Gpr171	4.19	3.19	4.09	4.15	4.83	1.99	1.92	4.22	4.61	No Peak	N/A	N/A	9.83	9.84	9.92	8.85	8.75	8.84	-1.05	0.00
Rab19	3.62	2.67	3.28	3.31	3.18	2.96	3.35	1.56	2.12	No Peak	N/A	N/A	7.74	7.80	7.71	6.65	6.53	6.53	-1.18	0.00
Atp8b4	4.92	4.71	5.77	4.82	5.56	5.11	4.38	5.81	5.94	No Peak	N/A	N/A	7.41	7.19	7.15	5.19	5.20	5.10	-2.09	0.00
Ttc13	4.16	3.82	3.48	3.53	2.91	0.64	2.99	3.08	2.66	No Peak	N/A	N/A	8.66	8.66	8.76	7.71	7.61	7.68	-1.02	0.00
Tnfrsf18	4.21	3.73	2.83	3.61	3.73	1.42	2.17	3.50	2.98	No Peak	N/A	N/A	9.96	10.06	9.98	8.80	8.67	8.65	-1.29	0.00
Stk38	3.13	3.57	2.52	1.42	2.69	2.64	2.18	1.62	3.45	No Peak	N/A	N/A	9.84	9.87	9.79	8.72	8.75	8.86	-1.06	0.00
Dnah8	0.71	1.01	2.88	1.41	2.29	1.84	2.11	2.31	-1.38	No Peak	N/A	N/A	9.48	9.49	9.55	7.28	7.26	7.44	-2.18	0.00
Tlr1	3.57	3.79	3.83	4.16	4.35	4.12	3.85	4.15	4.64	No Peak	N/A	N/A	7.60	7.46	7.49	5.73	5.50	5.77	-1.85	0.00
Tm6sf1	5.00	4.91	4.53	3.83	3.65	1.78	2.15	4.28	3.66	No Peak	N/A	N/A	8.93	8.94	8.84	7.79	7.85	7.94	-1.04	0.01
Ccdc69	4.58	3.14	3.85	3.64	5.27	3.39	2.04	4.40	4.14	No Peak	N/A	N/A	9.25	9.32	9.42	8.02	8.15	8.02	-1.27	0.01
Tmem71	2.91	4.40	4.38	3.78	4.56	3.23	3.97	4.26	4.84	No Peak	N/A	N/A	10.39	10.27	10.28	9.07	9.08	9.25	-1.18	0.01
Ly6c1	3.11	2.94	3.64	2.84	3.12	1.90	2.90	3.91	2.60	No Peak	N/A	N/A	10.63	10.59	10.21	7.85	7.70	7.53	-2.78	0.01
Ccr4	4.30	3.66	3.64	3.96	3.75	2.41	1.32	3.83	3.10	No Peak	N/A	N/A	9.95	10.31	10.49	7.25	7.14	7.40	-2.98	0.01
Itga4	3.28	4.21	3.87	4.10	4.68	2.62	-0.30	4.07	4.86	No Peak	N/A	N/A	11.30	11.28	11.31	9.78	9.84	10.06	-1.41	0.01
Inpp4a	4.33	3.48	3.60	2.75	3.18	3.62	3.55	4.01	3.61	No Peak	N/A	N/A	9.01	8.96	8.87	7.80	7.89	7.97	-1.06	0.01
Igflr1	3.69	2.65	2.92	2.07	1.82	1.98	3.01	1.60	1.12	No Peak	N/A	N/A	9.41	9.47	9.53	8.33	8.36	8.28	-1.15	0.00

Supplementary Table 2: Under-expressed genes.

The table lists the 115 genes ‘under-expressed’ in mature dKO OT-II CD4 SP thymocytes, as defined in Fig. 6c. Genes are ranked by decreasing H3K27Me3 signal ratio.

Column headings for Supplementary Tables 1 and 2

wt1, wt2, wt3 or dKO1, dKO2, dKO3 designate replicate samples processed for ChIPseq or microarray analyses.

FDR: False discovery rate.

Signal Ratio (-Log_2): Ratio of average H3K27Me3 signals in wild-type vs. dKO samples

Expression ratio (Log_2): Ratio of average microarray expression signals in dKO vs. wild-type samples.

Supplementary Methods

Primers used in this study

Primers for Chip-qPCR

Gene	Forward	Reverse
S1pr1	5'-agcccacgccatttccac-3'	5'-ttggtgccttctgtgagcaag-3'

Primers for RT-qPCR

Gene	Forward	Reverse
S1pr1	5'-cctctcggacctattagcaggc-3'	5'-cgcagaaaccactgggcag-3'
Klf2	5'-cggcacggatgaggacctaaac-3'	5'-tcgggaggattttcggcg-3'
Sell	5'-gcttccagtccaagtgtgctttc-3'	5'-tgaccagtttccagatgctccac-3'
Ccr4	5'-tctacagcggcatcttcttcat-3'	5'-cagtacgtgtggttgtgctctg-3'
Ccr7	5'-tacatcggcgagaataccacg-3'	5'-atgagaggcaggaaccaggc-3'
Ccnd2	5'-gagaagctgtctctgatccgc-3'	5'-cttccagttgcgatcatcgacg-3'
Irf4	5'-tcggcccaacaagctagaaa-3'	5'-ggccatggtgagcaaacact-3'
Lfng	5'-ctgcaccattggctacattg-3'	5'-atgggtcagcttccacagag-3'
Il6ra	5'-gcaagtccagccacaacg-3'	5'-actcgggtcccaggtctca-3'
Foxo1	5'-agatgagtgccctgggcagc-3'	5'-gatggactccatgtcacagt-3'
Nr4a1	5'-gcacagcttgggtgttgatg-3'	5'-cagacgtgacaggcagctg-3'
Cd69	5'-tggtcctcatcacgtccttaataa-3'	5'-tccaacttctcgtacaagcctg-3'
Gapdh	5'-gctgtgggcaaggtcatcc-3'	5'-gtttctccaggcggcacg-3'
18S rRNA	5'-aaatcagttatggttcctttggtc-3'	5'-gctctagaattaccacagttatccaa-3'

Primers for genotyping

Gene	Forward	Reverse
Jmd3	5'-gtgtcctggaacgtcgctcg-3'	5'-ggctggaggatggcagatg-3'
Utx	5'-gctggcactgttcattgggttc-3'	5'-ggcatactacctaagggtaggc-3'
Cre	5'-gcattgctgtcacttggtcgt-3'	5'-cgatgcaacgagtgatgagg-3'
P14	5'-gggtctctcctggtgttt-3'	5'-tctcctttctccgtgctg-3'
